# A revision of the octocoral genus *Ovabunda* Alderslade, 2001 (Anthozoa, Octocorallia, Xeniidae)

**DOI:** 10.3897/zookeys.373.6511

**Published:** 2014-01-23

**Authors:** Anna Halász, Catherine S. McFadden, Dafna Aharonovich, Robert Toonen, Yehuda Benayahu

**Affiliations:** 1 Department of Zoology, George S. Wise Faculty of Life Sciences, Tel Aviv University, Ramat Aviv, Tel Aviv 69978, Israel; 2 Department of Biology, Harvey Mudd College, 1250 N. Dartmouth Ave., Claremont, CA 91711, USA; 3 Hawai’i Institute of Marine Biology, University of Hawaii at Manoa, 46-007 Lilipuna Road, Kane’ohe, HI 96744, USA

**Keywords:** Red Sea, sclerite microstructure, taxonomy, *Xenia*

## Abstract

The family Xeniidae (Octocorallia) constitutes an abundant benthic component on many Indo-West Pacific coral reefs and is ecologically important in the Red Sea. The genus *Ovabunda* Alderslade, 2001 was recently established to accommodate previous *Xenia* species with sclerites comprised of a mass of minute corpuscle-shaped microscleres. The aim of the present study was to examine type material of *Xenia* species in order to verify their generic affiliation. We present here a comprehensive account of the genus *Ovabunda*, using scanning electron microscopy to depict sclerite microstructure. We assign three *Xenia* species to the genus: *O. ainex* **comb. n.**, *O. gohari* **comb. n.**, and *O. crenata* **comb. n.**; and synonymize several other species of *Ovabunda*. We provide a key to *Ovabunda* species and conclude that they are mainly confined to the Red Sea, with some occurrence in the West Indian Ocean.

## Introduction

Members of the octocoral family Xeniidae form a major component of shallow coral-reef communities in the tropical Indo-West-Pacific region, and in the Red Sea in particular (e.g., Gohar 1940; Benayahu 1990; Reinicke 1997). In the Red Sea, a remarkably high number of 34 species have been recorded from the family prior to this revision, 24 of which were originally described from the region (Reinicke 1995, 1997). Some of these species have not been reported in any other region, thus emphasizing their high diversity and importance in the Red Sea.

The xeniids comprise for the most part small and soft colonies, which are often slippery due to the secretion of large amounts of mucus (Fabricius and Alderslade 2001). A morphological feature unique to most of them is that the pinnules along the margins of the polyp tentacles are arranged in more than one longitudinal row. The number of pinnule-rows and the number of pinnules in the outermost row have been considered diagnostic features used for species identification (e.g., Hickson 1931a; Verseveldt and Cohen 1971; Benayahu 1990; Reinicke 1997). Several additional characteristics have been considered of taxonomic value, such as the size, shape, and coloration of the colonies, as well as polyp retractability and pulsation in live colonies (e.g., Gohar 1940; Verseveldt 1960; Reinicke 1995, 1997; Fabricius and Alderslade 2001).

Most xeniids feature a high density of sclerites in all parts of the colony, such as members of the genera *Asterospicularia* Utinomi, 1951; *Sansibia* Alderslade, 2000 (see Fabricius and Alderslade 2001) and *Xenia* including *Xenia blumi* Schenk, 1896; *Xenia garciae* Bourne, 1894 (see Gohar 1940) and *Xenia benayahui* Reinicke, 1995 (see Reinicke 1997); while other species have no sclerites or only a few (e.g., *Xenia hicksoni* Ashworth, 1899 and *Heteroxenia ghardaqensis* Gohar, 1940).

Over the years studies have revealed that a number of taxa have relatively simple sclerites in the form of round platelets, including those of the genera *Cespitularia* Milne-Edwards & Haime, 1850; *Heteroxenia* Kölliker, 1874; *Funginus* Tixier-Durivault, 1970; *Sansibia* Alderslade, 2000; *Sarcothelia* Verrill, 1928; *Sympodium* Ehrenberg, 1834 and *Xenia* Lamarck, 1816 (see also Fabricius and Alderslade 2001). This finding led to coining of the terms “general xeniid structure” (Gohar 1940: 95, 99, 104, 107) and “xeniid type of sclerites” (Verseveldt 1970: 227; Verseveldt 1974: 35). The majority of species descriptions of those genera thus did not depict sclerites, but rather presented the size range of their maximal diameter (e.g., *Xenia crassa* Schenk, 1896 in Hickson 1931a; *Xenia blumi* and *Xenia garciae* in Gohar 1940; *Xenia biseriata* Verseveldt & Cohen, 1971 and *Xenia faraunensis* Verseveldt & Cohen, 1971). Similarly, in his original description of *Xenia macrospiculata*, Gohar (1940) provided the size range of the sclerites and their morphological features, referring to them as “generally spherical, rarely ovoid or of irregular roughly spherical shape but always with smooth surface”; and also noted for *Xenia blumi* sclerites: “They are mostly ovoid, rarely circular or ellipsoidal”. Later, Fabricius and Alderslade (2001: 53) argued that xeniid sclerites are “nearly always minute platelets or corpuscle-like forms, with a surface that generally appears almost smooth at the magnification of a light microscope”. Only a few studies presented drawings of xeniid sclerites, such as those of *Cespitularia mantoni* Hickson, 1931a and *Cespitularia multipinnata* Quoy & Gaimard, 1833 (Hickson 1931a: 168, fig. 5); *Cespitularia taeniata* May, 1899 (Utinomi 1950: 15, fig. 3f); *Xenia kusimotoensis* Utinomi, 1955: 264, fig. 1d; *Fungulus heimi* Tixier-Durivault, 1970: 324; *Xenia macrospiculata* (see Verseveldt 1971: 64, fig. 39c), and *Sympodium caeruleum* Ehrenberg, 1834 (see Klunzinger 1877: 42, pl. III, fig. 5 from Ehrenberg’s unpublished drawings).

Over the last two decades the use of scanning electron microscopy (SEM) has revealed microstructural features of xeniid sclerites, which were not visible under a light microscope. Benayahu (1990) presented sclerite images of the type of *Xenia verseveldti* Benayahu, 1990; and of his collected *Xenia nana* Hickson, 1931b whose surface microstructure revealed corpuscular aggregations of microscleres, recorded for the first time among octocorals. Subsequently, SEM images with similar features were presented for *Xenia obscuronata* Verseveldt & Cohen, 1971 (Reinicke 1995: 18, fig. 6h, i), *Xenia benayahui* Reinicke, 1995: 18, fig. 6g and *Xenia faraunensis* Verseveldt & Cohen, 1971 (Reinicke 1997: 19, fig. 9a, b). A contrasting form, made of latticework matrix of calcite rods (*sensu* Alderslade 2001) was demonstrated for the first time by Reinicke (1997: 19, figs 7, 8, 10) for *Heteroxenia fuscescens* Ehrenberg, 1834; *Sympodium caeruleum* Ehrenberg, 1834 and *Xenia umbellata* Lamarck, 1816. It should be noted that the sclerites of *Heteroxenia fuscescens* and *Sympodium caeruleum* were initially depicted by Reinicke 1995 (page 18, fig. 6e, f), but unfortunately the micrographs were of low quality. Reinicke (1997) assigned the two types of sclerites, featuring both corpuscular and dendritic structure, to *Xenia*, and concluded that their taxonomic significance remained to be studied. These findings prompted the use of SEM for studying sclerites of the genera *Anthelia* Lamarck, 1816 (see Reinicke 1997) and *Asterospicularia* Utinomi, 1951 (see Alderslade 2001). The latter study further used SEM to describe the genera *Bayerxenia* Alderslade, 2001; *Ingotia* Alderslade, 2001; *Ixion* Alderslade, 2001 and *Orangaslia* Alderslade, 2001.

The discovery of a corpuscular sclerite-type among previously described *Xenia* species led Alderslade (2001) to establish the genus *Ovabunda* while retaining those with the dendritic surface in the original genus. Consequently, he assigned seven of the originally described *Xenia* species to the new genus. Those assignments were made based on examination of type colonies of *Xenia benayahui* and *Xenia verseveldti* by L.P. van Ofwegen and the last author of the current study. For the remaining species, the assignment was not based on examination of types, but rather on sclerite descriptions in Reinicke (1997) for *Xenia biseriata* Verseveldt & Cohen, 1971 (p. 18) and *Xenia faraunensis* (Fig. 9a, b), and in Reinicke (1995) for *Xenia obscuronata* (p. 18, fig. 6h, i). The assignment of *Xenia macrospiculata* was based on examination of Red Sea material by the last author of the current study (Alderslade 2001: fig. 30), and that of *Xenia arabica* Reinicke, 1995 was not justified by Alderslade.

In a later study, Janes (2008) depicted sclerites of *Ovabunda benayahui*, *Ovabunda hamsina* Reinicke, 1997 and *Ovabunda impulsatilla* Verseveldt & Cohen, 1971, as well as of his new species, *Ovabunda aldersladei*, all collected in the Seychelles and featuring *Ovabunda*-type sclerites with corpuscular microstructure. Aharonovich and Benayahu (2011) employed high-resolution environmental SEM (ESEM) for a study of sclerites of type material of *Ovabunda biseriata*, *Ovabunda faraunensis* and *Ovabunda impulsatilla*, which revealed how the microscleres adhere.

Establishment of the genus *Ovabunda* by Alderslade led us to examine type material that was originally described as *Xenia*, in order to verify its generic affiliation. Not only were the types of the species assigned to *Ovabunda* by Alderslade examined, and their sclerites studied using SEM, but the types of a number of other nominal species of *Xenia* were also studied (see Table 1). Consequently, the current study has assigned three *Xenia* species to *Ovabunda* and retained 20 in the former genus; it also synonymizes several other species and designates a neotype for *Ovabunda macrospiculata*. The findings of the study emphasize the importance of re-examination of type material and the use of SEM to study xeniid sclerite microstructure for taxonomic purposes.

**Table 1.** List of *Xenia* type material examined during the current study along with corresponding museum numbers.

Species name	Type	Museum and museum number
*Xenia actuosa* Verseveldt & Tursch, 1979	Holotype	RMNH Coel. 12866
*Xenia antarctica* Kükenthal, 1902	Syntype	MNHHWU 63
*Xenia bauiana* May, 1899	Syntype	ZMB 3673
*Xenia blumi* Schenk, 1896	Holotype	SMF 44
*Xenia crassa* Schenk, 1896	Holotype	SMF 39
*Xenia delicata* Roxas, 1933	Syntype	ZMB 6908
*Xenia fusca* Schenk, 1896	Syntype	SMF 40
*Xenia garciae* Bourne, 1894	Type	BML 1921.11.18.1
*Xenia grasshoffi* Verseveldt, 1974	Holotype	SMF 2616
*Xenia kükenthali* Roxas, 1933	Holotype	ZMB 6917
*Xenia lepida* Verseveldt, 1971	Holotype	RMNH Coel. 6703
	Paratype	RMNH Coel. 6704
*Xenia membranacea* Schenk, 1896	Holotype	SMF 41
*Xenia mucosa* Verseveldt & Tursch, 1979	Holotype	RMNH Coel. 12867
*Xenia multispiculata* Kükenthal, 1909	Syntype	ZMB 6920
*Xenia novaebritanniae* Ashworth, 1900	Type	BML 1962.7.20.148
	Syntype	BML 1962.7.20.149
*Xenia plicata* Schenk, 1896	Holotype	SMF 45
*Xenia rubens* Schenk, 1896	Holotype	SMF 46
*Xenia sansibariana* May, 1899	Syntype	ZMB 3828
*Xenia ternatana* Schenk, 1896	Holotype	SMF 43
*Xenia viridis* Schenk, 1896	Holotype	SMF 42

## Methods

The study examined ethanol-preserved type specimens obtained on loan from the British Museum of Natural History (BML); National History collections of the Hebrew University of Jerusalem (HUJ); Museum of Natural History, Wroclaw University, Poland (MNHHWU); The Naturhistorisches Museum Wien (NHMW); the Naturalis Biodiversity Centre, formerly Rijksmuseum van Natuurlijke Historie, Leiden (RMNH); Senckenberg Naturmuseum Frankfurt (SMF); Zoologisches Museum Berlin (ZMB); and the Zoological Museum of Tel Aviv University (ZMTAU). Neotype material of *Ovabunda macrospiculata* was collected by the last author from the northern Red Sea, Gulf of Suez, Shag Rock from ~5 meters, 11 July 1986 and preserved in 70% ethanol.

**Figure 1.**
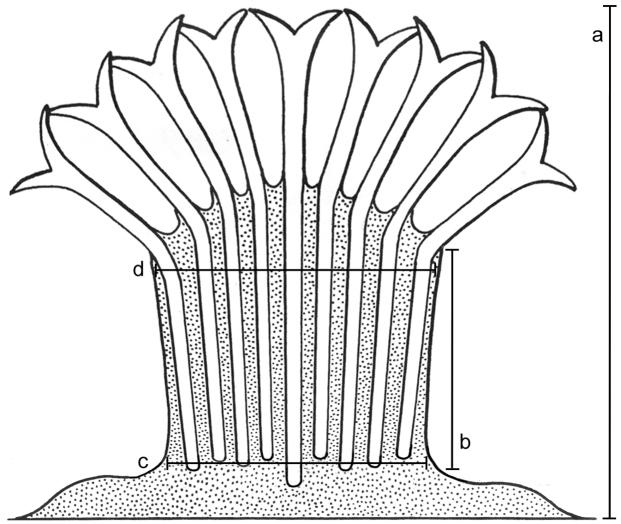
Illustration of colony dimensions. **a** Colony height **b** Stalk length **c** Stalk width at base **d** Stalk width at uppermost part. Illustration adopted from Hickson (1931a).

**Figure 2.**
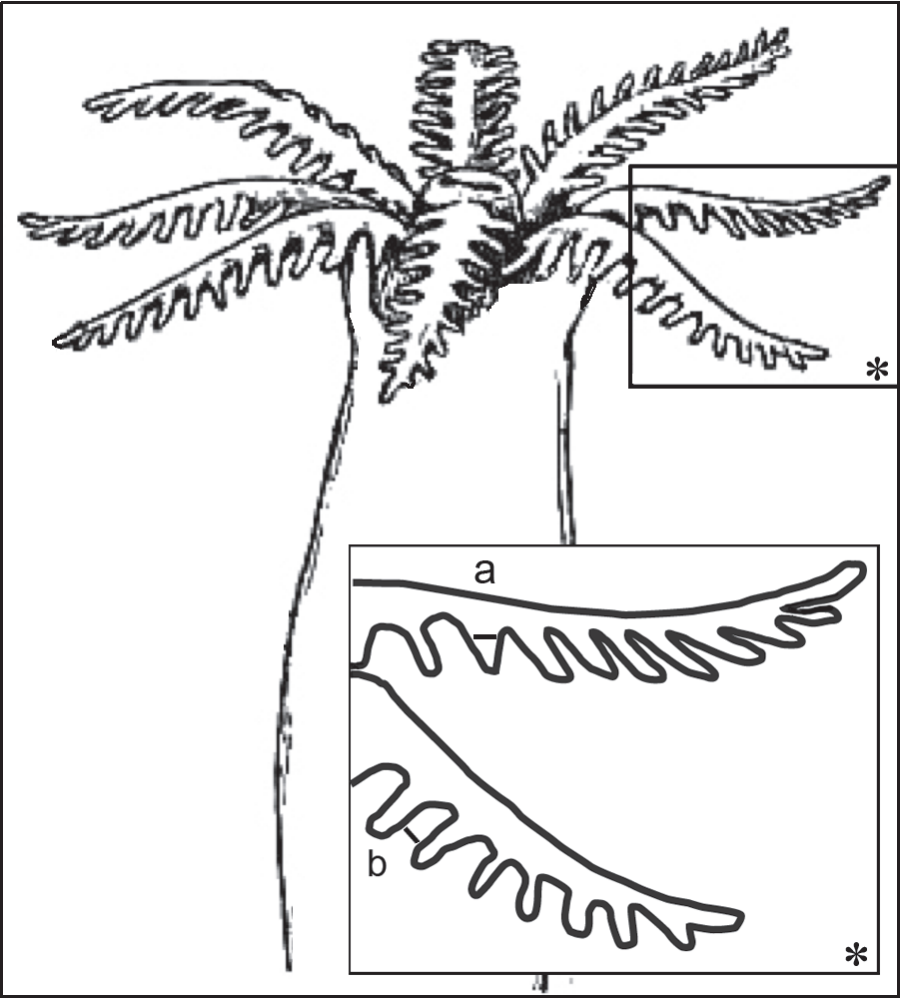
Illustration of polyp dimensions. **a** Pinnule width at its base **b** Gap between adjacent pinnules; asterisk indicates magnified area. Illustration adopted from Encyclopedia Britannica, 11th Edition, Volume 3 (http://www.digilibraries.com/html_ebooks/105312/34018/www.digilibraries.com@34018@34018-h@34018-h-7.htm).

Morphological features of the preserved colonies were recorded: colony height, numbers of branches, stalk length, width at colony-base, and width at the uppermost part below the capitulum where the polyps arise (Fig. 1). The number of rows of pinnules and number of pinnules in the outermost row were counted under a compound microscope from five to ten tentacles, as much as possible from different polyps. The polyps examined were the biggest within the colony and preferably from the mid-part of the capitulum, and not from the outer part which contains the newly grown and smaller polyps. In addition, the length of the anthocodiae – consisting of the polyp body and extended tentacles – the dimensions of the pinnules (length and width at base), and the distance between adjacent pinnules were recorded (Fig. 2).

To examine the sclerites, the tissue was treated with 10% sodium hypochlorite followed by repeated rinses in distilled water. Wet preparations of the clean sclerites from polyps, colony stalk, the coenenchyme and canal walls within were examined and photographed under an Optishot Nikon light microscope at × 400 magnification. After comparison of these sclerites we concluded that there are no differences in appearance and dimensions between sclerites in different colony parts. Therefore, only polyp sclerites were examined under SEM and presented in this paper. SEM stubs for polyp sclerites were prepared following Aharonovich and Benayahu (2011), and examined with a Jeol 840Å electron microscope. Measurements of sclerite images, taken by both light microscopy (images not presented) and SEM, were carried out using ImageJ 1.440 (National Institute of Health, USA). At least 20 randomly selected sclerites were measured for each colony in order to determine sclerite size variation. Number of sclerites measured is indicated in the text for each species. Since the sclerites are mostly spheroids, their dimensions are presented as the range of their minimal-to-maximal smallest diameters × range of their minimal-to-maximal largest diameters. The current study undertakes to clarify some confusion that resulted from differences between Reinicke (1995) and Reinicke (1997) which can be found in some descriptions in the Remarks section (e.g. *Ovabunda crenata*, *Ovabunda hamsina*).

The zoogeographical species distribution is based on the examination of types and other material, unless specified otherwise in the text. Table 1 lists *Xenia* types that were examined by us and maintained their original generic affiliation.

## Results

### Systematic section
Order Alcyonacea Lamouroux, 1812

#### Family Xeniidae

Ehrenberg, 1828

http://species-id.net/wiki/Xeniidae

Ovabunda Genus Alderslade, 2001: 49–52.

##### Diagnosis.

Colonies are small and soft with cylindrical stalks, undivided or branched, terminating in one or more domed polyp-bearing regions. Polyps are not retractile and are always monomorphic. Sclerites are oval spheroids, usually abundant in all parts of the colony, mostly up to 0.035 mm in maximal diameter, and comprised of a mass of minute corpuscle-shaped microscleres.

##### Key to species

**Table:** 

1	**Non-pulsating polyps in live colonies**	
1.1	**One row of pinnules on each side of the tentacles**	
	6–7 pinnules in each row	*Ovabunda benayahui*
	12–18 pinnules in each row	*Ovabunda verseveldti* (No data on pulsation present in the literature)
1.2	**Mostly one row, but sometimes two rows of pinnules on each side of the tentacles**	
	18–22 pinnules in the outermost row	*Ovabunda gohari*
1.3	**Two rows of pinnules on each side of the tentacles**	
	8–11 pinnules in the outermost row	*Ovabunda impulsatilla*
	13–16 pinnules in the outermost row	*Ovabunda biseriata*
	17–24 pinnules in the outermost row	*Ovabunda faraunensis*
	24–29 pinnules in the outermost row	*Ovabunda arabica*
1.4	**Mostly two, but sometimes three rows of pinnules in the outermost row**	
	12–16 pinnules in the outermost row	*Ovabunda crenata*
1.5	**Three rows of pinnules on each side of the tentacles**	
	15–20 pinnules in the outermost row	*Ovabunda ainex*
1.6	**Mostly three but sometimes four rows of pinnules in the outermost row**	
	17–22 pinnules in the outermost row	*Ovabunda hamsina*
2	**Pulsating polyps in live colonies**	
2.1	**Three rows of pinnules on each side of the tentacles**	
	14–18 pinnules in the outermost row	*Ovabunda macrospiculata*

#### Ovabunda ainex

(Reinicke, 1997) comb. n.

http://species-id.net/wiki/Ovabunda_ainex

Fig. 3

Xenia ainex Reinicke, 1997: 45–48, figs 19a–b, plates 6, 26.

##### Material.

**Holotype:** RMNH Coel. 23539, Sudanese Red Sea, Sanganeb Atoll, 20 km off Port Sudan, southern-slope near jetty (19°21'33.81"N, 37°19'37.66"E), 6 m, April 1991, coll. G. B. Reinicke; **paratype:** RMNH Coel. 23540, same location, 5 m S- jetty, October 1992, coll. G. B. Reinicke; **additional material:** RMNH Coel. 23535, Red Sea, Gulf of Aqaba, Aqaba, Saudi Arabian border Bay (29°21'37.31"N, 34°57'39.48"E), October 1989, coll. G. B. Reinicke; NHMW 2250, Saudi Arabia; **holotype of *Xenia crassa*** SMF 39, Indonesia, Ternate island, 1894, coll. Kükenthal; **holotype of *Xenia ternatana*** SMF 43, Indonesia, Ternate island, 1894, coll. Kükenthal.

##### Description.

The holotype is 45 mm high; its stalk is 15 mm long and splits at the base into three branches, two of which split again into two, 22 and 18 mm above the colony base; the latter branches are 35 and 30 mm long, 8 and 10 mm wide at their base, 4 and 6 mm wide at their uppermost part, respectively. The third branch splits 20 mm above the base into three branches, and is 40 mm long and 7 mm wide at its base and 5-7 mm wide at the uppermost part. The polyp’s body is up to 10 mm long, and the tentacles are up to 5 mm long, featuring three rows of pinnules on each side. The pinnules are relatively slender, up to 1 mm long and 0.08 mm wide, 17-20 in the outermost row with a space of one to two pinnule widths between adjacent pinnules. The spheroidal sclerites, typical regular *Ovabunda* sclerites, are scarce in all parts of the colony, measuring 0.011–0.030 × 0.018–0.042 mm in diameter (n = 25), composed of a mass of corpuscle-shaped microscleres. The original description indicated non-pulsating polyps for the live colonies. The ethanol-preserved holotype is light yellowish-beige, almost transparent.

**Figure 3.**
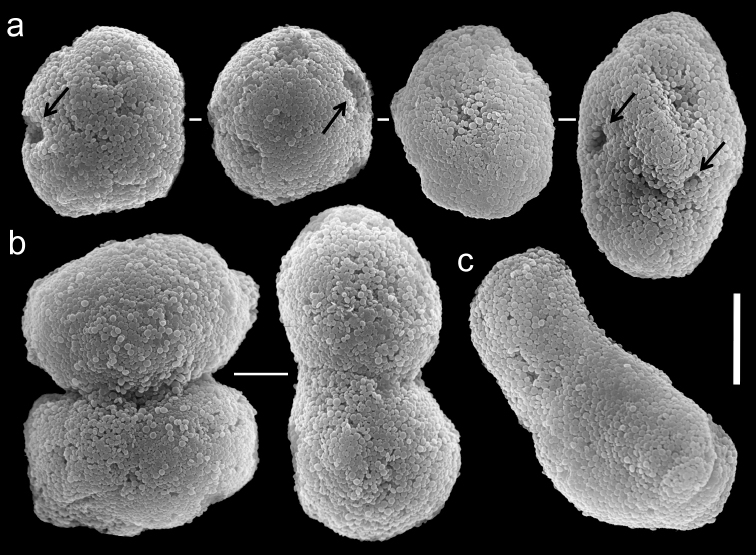
Scanning electron micrographs of polyp sclerites of *Ovabunda ainex* (Reinicke, 1997) paratype (RMNH Coel. 23540). **a** Regular sclerites **b** Fused sclerites **c** Irregular sclerite. Arrows indicate surface dents. Scale bar: 10 µm.

The paratype is 20 mm long; its stalk is 15 mm wide at its base, splitting into two branches, each 5 mm long and 10 and 8 mm wide at the base. The polyp’s body is up to 5 mm long, and the tentacles up to 5 mm long, bearing three rows of pinnules and 15–17 pinnules in the outermost row. The pinnules are 1.6 mm long and 0.16 mm wide, with a gap between adjacent pinnules ranging from one to three pinnule widths. The sclerites are *Ovabunda vabunda*-type, regular or irregular in shape (Fig. 3a, c), 0.012–0.026 × 0.02–0.04 mm in diameter (n = 21 sclerites). When two sclerites fuse they reach 0.039 mm in maximum diameter (Fig. 3b). The original description indicated non-pulsating polyps for live colonies. The ethanol-preserved colony is light beige.

##### Remarks.

RMNH Coel. 23535 features polyps with three rows of pinnules on each side of the tentacles and 16–19 pinnules in the outermost row. The sclerites are *Ovabunda*-type, measuring 0.016–0.026 × 0.022–0.030 mm in diameter (n =13).

Reinicke (1997) noted under the species *Xenia ainex* n.sp. (p. 44): “nec *Xenia ternatana*; Kükenthal 1913: 8 (partim. NHMW 2250)” and “*Xenia crassa*; Reinicke 1995: 43, Fig. 32”. The description of *Xenia ternatana* given by Kükenthal (1913) indicated two rows of pinnules, with 18 pinnules on average, and sclerites measuring 0.017 mm in diameter, thus differing from the features of *Ovabunda ainex* (see above). The NHMW 2250 specimen of *Xenia ternatana* was examined and found to match *Ovabunda ainex*, as stated by Reinicke (1997). *Xenia crassa* was suggested to be a synonym of *Ovabunda ainex* (Reinicke 1997). The current examination of the types of *Xenia crassa* and *Xenia ternatana* indicates that their sclerites distinctly differ from those of *Ovabunda ainex*, and thus those species’ original generic assignment should be retained.

##### Conclusions.

Our findings confirm the original description of Reinicke (1997) of *Ovabunda ainex*, but the structure of its sclerites justifies the generic re-assignment of the species to *Ovabunda*.

##### Similar species.

*Ovabunda ainex* is most similar to *Ovabunda macrospiculata*. Although they both have three rows of pinnules, the number of pinnules in the outermost row ranges from 15–20 in *Ovabunda ainex* compared to 14–18 in *Ovabunda macrospiculata*. The major difference between them, however, is that *Ovabunda macrospiculata* has pulsating polyps in live colonies and *Ovabunda ainex* does not.

##### Distribution.

Red Sea: Gulf of Aqaba, Sudan.

#### Ovabunda arabica

(Reinicke, 1995)

http://species-id.net/wiki/Ovabunda_arabica

Figs 4 ,5

Xenia arabica Reinicke, 1995: 37, figs 47–49; 1997: 36, fig. 14.
Ovabunda arabica ; Alderslade 2001: 51.
Xenia crista Reinicke, 1997: 38, figs 16a–b, plate 23, **syn. n.**

##### Material.

**Holotype:** RMNH Coel. 18673, northern Red Sea, Gulf of Aqaba, Saudi Arabian border bay, 20 km South of Aqaba (29°21'37.31"N, 34°57'39.48"E), 15 m, 12 November 1991, coll. G. B. Reinicke. **Paratypes:** RMNH Coel. 18675, northern Red Sea, Gulf of Aqaba, Nature Reserve (Aqaba), Marine science station (MSS), 10 km South of Aqaba (29°27'27.33"N, 34°58'24.19"E), 15 m, 4 November 1991, coll. G. B. Reinicke; RMNH Coel. 18676, location as above, 12 m, 5 November 1991, coll. G. B. Reinicke; **additional material: holotype of *Xenia crista*** RMNH Coel. 18677, northern Red Sea, Gulf of Aqaba, Nature Reserve (Aqaba), Marine science station (MSS), 10 km South of Aqaba (29°27'27.33"N, 34°58'24.19"E), 12 m, October 1989, coll. G. B. Reinicke; **paratype of *Xenia crista*** RMNH Coel. 18678, location as above, 15 m, October 1990, coll. G. B. Reinicke.

##### Description.

The holotype is 45 mm high; its stalk is 15 mm long, 7 mm wide at its base and 7 mm wide at its upper part. The polyp’s body is 12 mm long, and tentacles up to 11 mm long. The pinnules are 2 mm long and 0.2 mm wide at the base; the gap between adjacent pinnules is one pinnule wide. There are two rows of pinnules on each side and 24-25 pinnules in the outermost row. The sclerites are spheroids of the *Ovabunda*-type, measuring 0.028–0.036 mm at largest diameter.

**Figure 4.**
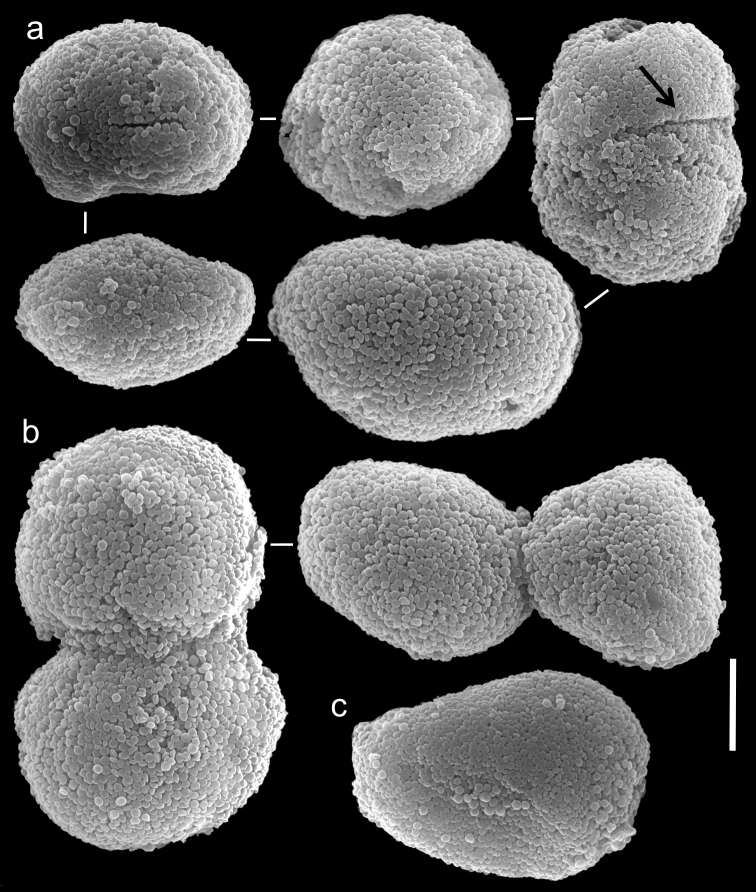
Scanning electron micrographs of polyp sclerites of *Ovabunda arabica* (Reinicke, 1995) paratype (RMNH Coel. 18675). **a** Regular sclerites **b** Fused sclerites **c** Pear-shaped sclerite. Arrow indicates surface irregularity, might represent the fusion area of two individual sclerites. Scale bar 10 µm.

One paratype (RMNH Coel. 18675) has a total height of 20 mm; its stalk is 16 mm long, 10 mm wide at the base, and 18 mm wide at its uppermost part. The polyp’s body is 9 mm long; the tentacles are 6 mm long, featuring two rows of pinnules 2.2 mm long and 0.2 mm wide on each side, and 24–29 pinnules in the outermost row. The pinnules mostly feature a one pinnule width gap. Sclerites are *Ovabunda*-type, regular and pear- shaped (Fig. 4a, c), measuring 0.016–0.030 × 0.022–0.038 mm in diameter (n = 27 sclerites). Occasionally, two sclerites are fused (Fig. 4b), measuring up to 0.048 mm in diameter. The sclerites are more abundant on the aboral side of the tentacles than the oral one. The second paratype (RMNH Coel. 18676) has 2 rows of pinnules on each side of the tentacles, 24–27 in the outermost row. The sclerites are *Ovabunda*-type, measuring 0.026–0.036 mm at maximal diameter. The original description indicated non-pulsating polyps for live colonies. Colour of the ethanol-preserved holotype is light brown.

##### Remarks.

The number of rows of pinnules and pinnules in the outermost row correspond to the original description of the holotype and paratypes of *Xenia arabica*. Their sclerites are *Ovabunda*-type (Fig. 4) and, therefore, the species should be reassigned to that genus. Sclerite sizes given for the holotype and paratype in the original description exceed those obtained by us, as follows: holotype 0.043–0.053 mm *vs.* 0.028–0.036 mm at the larger diameter, and paratypes (RMNH Coel. 18675) 0.039 × 0.043 *vs.* 0.016–0.030 × 0.022–0.038 mm and (RMNH Coel. 18676) 0.043 × 0.051 *vs.* 0.026–0.036 mm, respectively. The larger size of 0.053 and 0.051 mm indicated by Reinicke (1997) falls within that of other *Ovabunda* species, such as *Ovabunda crenata* for the fused sclerites and *Ovabunda gohari* for larger and rare individual sclerites.

**Figure 5.**
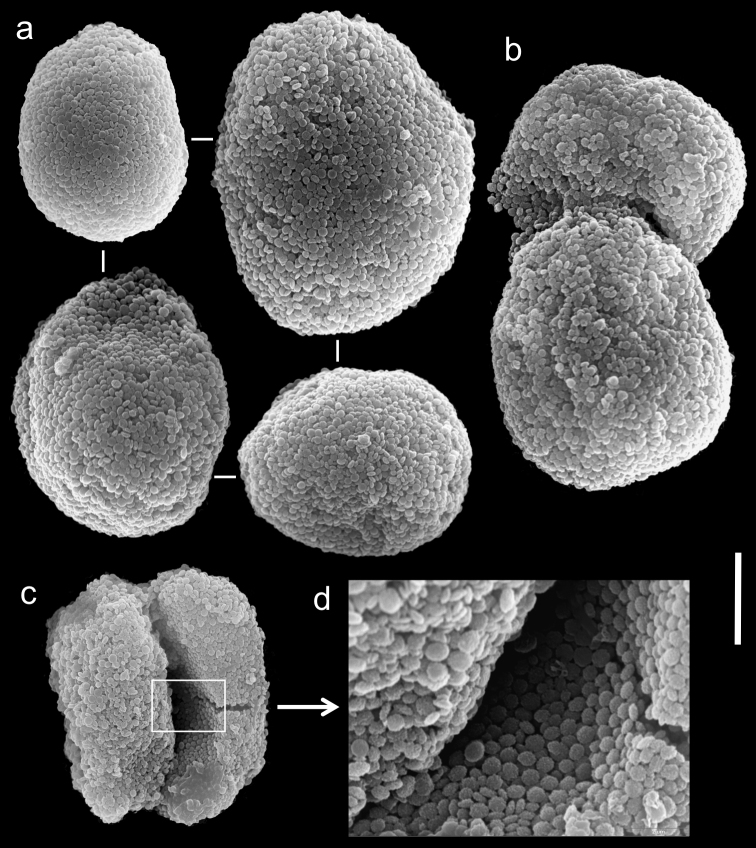
Scanning electron micrographs of polyp sclerites of *Xenia crista* Reinicke, 1997 holotype (RMNH 18677). **a** Regular sclerites **b–c** Fused sclerites **d** White rectangle in c indicates magnified area. Scale bar 10 µm.

The holotype of *Xenia crista* (RMNH Coel. 18677) is 50 mm high and its stalk is 27 mm long, split into two branches 17 mm above its base. The branches are 10 mm long and 8 mm wide at their base and uppermost part. The polyp’s body is up to 10 mm long. The tentacles are up to 10 mm long; pinnules up to 1.5–2 mm long and 0.2 mm wide, featuring one pinnule-width gap between adjacent pinnules. The tentacles have two rows of pinnules on each side, with 22–30 pinnules in the outermost row. Sclerites are *Ovabunda*-type (Fig. 5a), measuring 0.014–0.030 × 0.017–0.037 mm in diameter (n = 48 sclerites). Occasionally, two sclerites are fused, reaching a maximal diameter of 0.042 mm (Fig. 5b, c).

The paratype of *Xenia crista* (RMNH Coel. 18678) features tentacles with two rows of pinnules and 26–29 pinnules in the outermost row. The sclerites are *Ovabunda*-type, 0.025–0.036 mm in maximal diameter. The original description of the species indicated non-pulsating polyps in live colonies.

##### Conclusions.

Both the original description of *Xenia crista* and the current examination revealed two rows of pinnules; however, we found 22–30 pinnules in the holotype and 26–29 in the paratypes, compared to 29–33 and 28–32, respectively, in the original description. The taxonomic features of *Xenia crista* overlap those of *Ovabunda arabica* and, therefore, they should be considered as synonyms, giving an alphabetical priority to *Ovabunda arabica*.

##### Similar species.

*Ovabunda arabica* is most similar to *Ovabunda faraunensis*. Although they both have two rows of pinnules and both have non-pulsating polyps in living colonies, the number of pinnules in the outermost row ranges from 24–29 in *Ovabunda arabica* compared to 17–24 in *Ovabunda faraunensis*.

##### Distribution.

Red Sea: Gulf of Aqaba.

#### Ovabunda benayahui

(Reinicke, 1995)

http://species-id.net/wiki/Ovabunda_benayahui

Figs 6 ,7

Xenia nana ; Benayahu 1990: 117–118, fig. 3, table 1.
Xenia benayahui Reinicke, 1995: 26–27, figs 1c, 15; 1997: 29, fig. 12, plate 16.
Ovabunda benayahui ; Alderslade 2001: 51; Janes 2008: 609–610, fig. 7.

##### Material.

**Holotype and two paratypes**(the holotype is the largest colony): RMNH Coel. 19664, northern Red Sea, Gulf of Aqaba, Saudi Arabian border bay, 20 km south to Aqaba (29°21'37.31"N, 34°57'39.48"E), 21 m, 3 October 1989, coll. G. B. Reinicke; **additional material:** ZMTAU Co 26043, northern Red Sea, Gulf of Suez, Shag Rock (27°47'13.59"N, 34°0'22.61"E), 14 July 1987, coll. Y. Benayahu; ZMTAU Co 26044, northern Red Sea, Gulf of Suez, Southern tip of Sinai, Shaab el Utaf (27°45'9.00"N, 34°10'18.00"E), 10 m, 8 July 1986, coll. Y. Benayahu.

##### Description.

**Figure 6.**
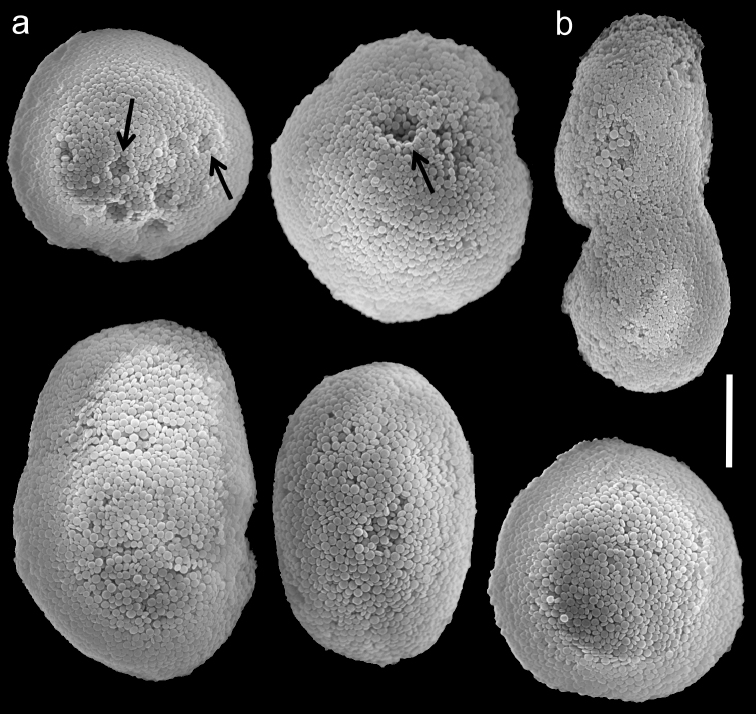
Scanning electron micrographs of polyp sclerites of *Ovabunda benayahui* (Reinicke, 1995) holotype (RMNH Coel. 19664). **a** Regular sclerites **b** Fused sclerite. Arrows indicate surface dents. Scale bar 10 µm.

The holotype is 25 mm high; stalk is 10 mm long, 6 mm wide at colony-base, and 15 mm wide at the uppermost part. The polyp’s body is up to 4 mm long, and the tentacles up to 4 mm long, featuring a single row of 6–7 pinnules along each edge. The pinnules are short, up to 0.7 mm long and 0.4 mm wide with a 0.2–0.3 mm gap between adjacent pinnules. The sclerites are *Ovabunda*-type spheroids (Fig. 6a) and measure 0.019–0.035 × 0.027–0.041 mm in diameter (n = 22 sclerites). When two sclerites are fused they measure up to 0.043 mm in maximal diameter (Fig. 6b). The original description indicated non-pulsating polyps in live colonies. The ethanol-preserved colony is light brown-beige.

##### Remarks.

**Figure 7.**
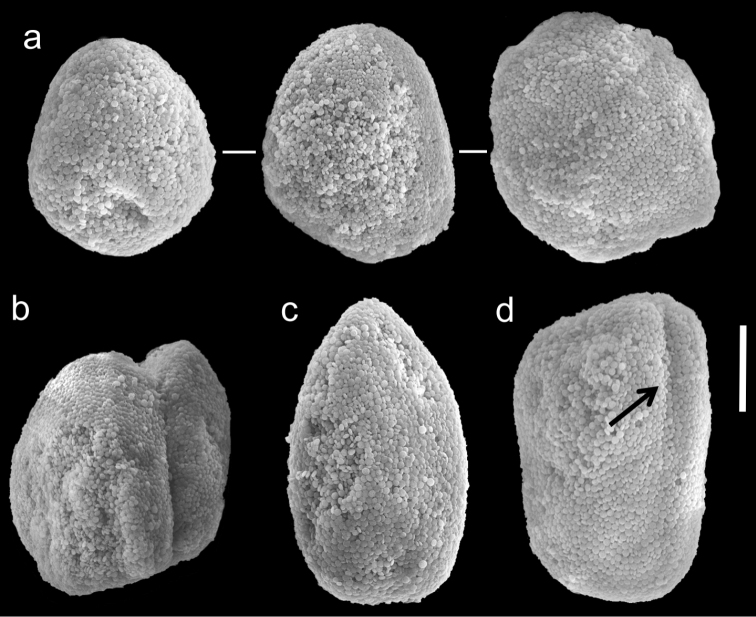
Scanning electron micrographs of polyp sclerites of *Ovabunda benayahui* Reinicke, 1995 (ZMTAU Co 26043). **a** Regular sclerites **b** Fused sclerites **c** Egg-shaped sclerite **d** Rectangular sclerite. Arrow indicates surface crest. Scale bar 10 µm.

ZMTAU Co 26043, originally identified by Benayahu (1990) as *Xenia nana*, comprises 13 colonies up to 17 mm in height. Their stalks are not divided, and are up to 8 mm long, 8 mm wide at the base, and 10 mm wide at the uppermost part. Polyp’s body reaches up to 1.4 mm in length, tentacles up to 1.6 mm, featuring a row of 5–6 closely set pinnules, 0.4 mm long and 0.16 mm wide. Sclerites are *Ovabunda*-type spheroids (Fig. 7a), 0.016–0.029 × 0.020–0.039 mm in diameter (n = 34 sclerites); some are egg-shaped (Fig. 7c) and sometimes two spheroids are fused (Fig. 7b). ZMTAU Co 26044 comprises several disintegrated small colonies, similar in size to those of ZMTAU Co 26043. There are 5–7 pinnules in a single row on each side of the tentacles. The sclerites are *Ovabunda*-type, 0.015–0.031 × 0.022–0.039 mm in diameter (n = 34 sclerites). Polyp pulsation was not noted by Benayahu (1990). The colonies (ZMTAU Co 26043–26044) were misidentified as *Xenia nana* by Benayahu (1990) and should be reassigned to *Ovabunda benayahui*. The type of *Xenia nana* (BML 1939.6.12.9) was examined by the last author of the current paper and found to be of the genus *Aldersladum* Benayahu & McFadden, 2011.

##### Conclusions.

The features of the holotype and paratypes of *Ovabunda benayahui* agree with the original description of the species, and the assignment to *Ovabunda* by Alderslade (2001) was confirmed in the current study.

##### Similar species.

*Ovabunda benayahui* is most similar to *Ovabunda verseveldti*. Although they both have one row of pinnules, the numbers of pinnules in the outermost row ranges from 6–7 in *Ovabunda benayahui* compared to 12–18 in *Ovabunda verseveldti*.

##### Distribution.

Red Sea: Gulf of Aqaba, Southern tip of Sinai Peninsula; Seychelles (see Janes 2008).

#### Ovabunda biseriata

(Verseveldt & Cohen, 1971)

http://species-id.net/wiki/Ovabunda_biseriata

Figs 8 ,9

Xenia biseriata Verseveldt & Cohen, 1971: 60, table 1; Benayahu 1990 table 1 listed only; Reinicke 1997: 33, plate 20.
Xenia obscuronata Verseveldt & Cohen, 1971: 60, table 1, fig 10; Benayahu 1990 table 1 listed only; Reinicke 1997: 33, 35, plates 2, 4, 7, 21.
Ovabunda biseriata ; Alderslade 2001: 51; Aharonovich and Benayahu 2011.
Ovabunda obscuronata ; Alderslade 2001: 5, **syn. n.**

##### Material.

**Holotype and five paratypes:** HUJ I Co. 72, northern Red Sea, Gulf of Aqaba, Marsa Murach (29°25'34.44"N, 34°50'10.46"E), 1–4 m, 15 September 1969, coll. Y. Cohen; **holotype of *Ovabunda obscuronata*** HUJ I Co. 120, northern Red Sea, Gulf of Aqaba, Ras el Muqebla (29°24'1.20"N, 34°48'41.99"E), 12 m, 16 August 1971, coll. Y. Cohen; **holotype of *Xenia ternatana*** SMF 43, Indonesia, Ternate island, 1894, coll. Kükenthal.

##### Description.

**Figure 8.**
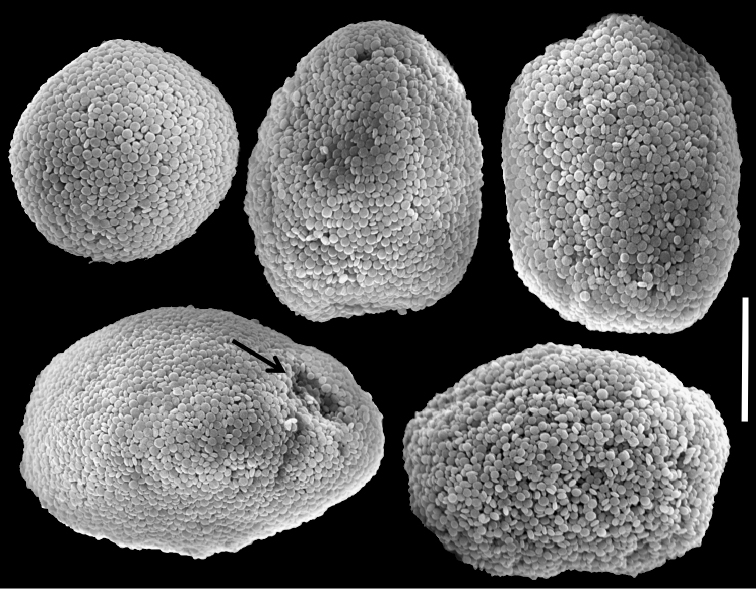
Scanning electron micrographs of polyp sclerites of *Ovabunda biseriata* (Verseveldt & Cohen, 1971) holotype (HUJ I Co. 72). Arrow indicates surface dents. Scale bar 10 µm.

The holotype is 28 mm high, stalk is 15 mm long, 11 mm wide at its base and 10 mm wide at the uppermost part; it is attached to the skeleton of a stony coral. Polyp’s body reaches up to 8–10 mm, and the tentacles 6–7 mm, bearing pinnules up to 1 mm long and 0.24 mm wide, separated by less than a pinnule-width. Two rows of pinnules are aligned along each of the tentacles margins, with 13–15 pinnules in the outermost row. There are numerous sclerites, abundant in all parts of the colony except for the mid-line of the oral side of the tentacles, where they are scarce. The sclerites are *Ovabunda*-type (Fig. 8), measuring 0.013–0.029 × 0.018–0.039 mm in diameter (n = 100 sclerites). Rarely, two sclerites are fused, reaching a maximum diameter of 0.048 mm and occasionally 0.060 mm. The paratypes are of similar size to the holotype, featuring tentacles with two rows of pinnules and 13–16 pinnules in the outermost row. The stalk of the smallest paratype is divided into two branches; the paratype tentacles bear two rows of pinnules with 10–12 pinnules in the outermost row. The sclerites of all paratype colonies are *Ovabunda*-type, ranging 0.018–0.025 × 0.023–0.039 mm in diameter. The original description of the species indicated non-pulsating polyps in live colonies. The preserved colonies are yellowish-light beige in colour.

##### Remarks.

**Figure 9.**
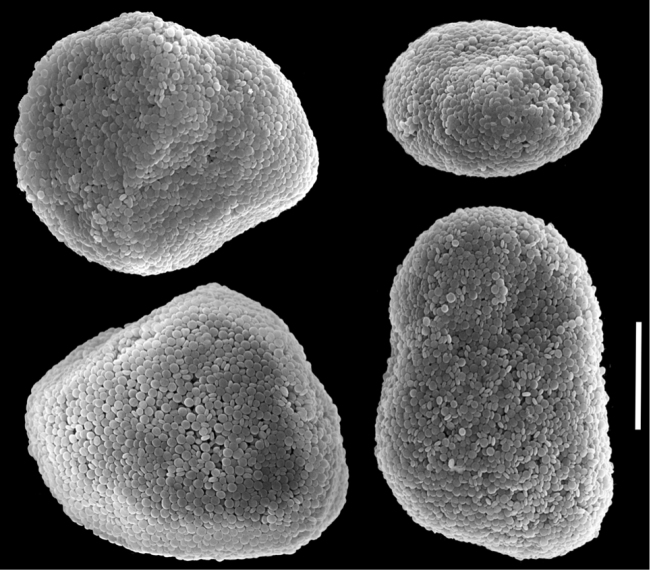
Scanning electron micrographs of polyp sclerites of *Ovabunda obscuronata* (Verseveldt & Cohen, 1971) holotype (HUJ I Co. 120). Scale bar 10 µm.

In general the original description of the species corresponds to the current findings. SEM micrographs of the holotype sclerites (Fig. 8) indicate that they are indeed *Ovabunda*-type, and therefore further confirm the previous assignment to that genus (Alderslade 2001; Aharonovich and Benayahu 2011). The number of rows of pinnules, the number of pinnules in the outermost row, and the dimensions of the holotype colony of *Ovabunda obscuronata* (HUJ I Co. 120) correspond to the original description by Verseveldt and Cohen (1971). The sclerites are oval spheroids, *Ovabunda*-type, ranging 0.012–0.026 × 0.018–0.041 mm in diameter (Fig. 9, n = 61 sclerites). Rarely, two sclerites are fused, reaching a maximal diameter of 0.060 mm. The original description indicated a maximum diameter of 0.045 mm for the stalk sclerites, 0.060 mm for those of the anthocodiae and “irregular spicules” in the tentacles (Verseveldt and Cohen 1971: 60). The latter probably referred to fused sclerites, which were not identifiable under light microscopy. The original description of *Ovabunda obscuronata* indicated non-pulsating polyps in live colonies, similar to *Ovabunda biseriata*. The ethanol-preserved holotype is light brown in colour.

Reinicke (1997) noted under the description of *Xenia obscuronata* (p. 33): “nec *Xenia ternatana*; Kükenthal 1913: 8 (in part)”. The type of *Xenia ternatana* was examined during the present study and its features do not agree with those of *Ovabunda obscuronata*. *Xenia ternatana* features platelets composed of dendritic rods, measuring up to 0.022 mm in maximal diameter. It should be noted that the description of *Xenia ternatana* by Kükenthal also does not correspond to the features of *Ovabunda obscuronata*.

##### Conclusions.

Examination of the holotype of *Ovabunda biseriata* and *Ovabunda obscuronata* revealed certain similarities, as already noted in the original description (Verseveldt and Cohen 1971): two rows of pinnules, with 12–15 *vs.* 14–16 pinnules, respectively, in the outermost row. The original description noted differences in the size of sclerites; 0.018–0.035 mm in *Ovabunda biseriata* vs. 0.045 mm in the stalk and anthocodiae and 0.060 mm in the tentacles of *Ovabunda obscuronata*. The current examination revealed a similar size range in the two species (0.013–0.029 × 0.018–0.039 *vs.* 0.012–0.026 × 0.018–0.041, respectively). Therefore, it is concluded that *Ovabunda biseriata* and *Ovabunda obscuronata* are in fact synonyms, giving alphabetical priority to the former.

##### Similar species.

*Ovabunda biseriata* is most similar to *Ovabunda impulsatilla* and *Ovabunda faraunensis*. Although they all have two rows of pinnules and non-pulsating polyps in living colonies, the number of pinnules in the outermost row ranges from 13–16 in *Ovabunda biseriata* compared to 8–11 in *Ovabunda impulsatilla* and 17–24 in *Ovabunda faraunensis*.

##### Distribution.

Red Sea: Gulf of Aqaba, Gulf of Suez, Sudan.

#### Ovabunda crenata

(Reinicke, 1997) comb. n.

http://species-id.net/wiki/Ovabunda_crenata

Fig. 10

Xenia crenata Reinicke, 1997: 41–42, figs 3c, 15; plates 5, 25.

##### Material.

**Holotype:** RMNH Coel. 23538, Sudanese Red Sea, Sanganeb Atoll, 20 km off Port Sudan, S-slope near jetty (19°21'33.81"N, 37°19'37.66"E), 10 m, April 1991, coll. G.B. Reinicke; **additional material:** RMNH Coel. 23517, Sudanese Red Sea, Sanganeb Atoll, lagoon slope, TQ II station, 8 m, March 1991, coll. G.B. Reinicke; **type of *Xenia viridis*** SMF 42, Indonesia, Ternate island, 1894, coll. Kükenthal; **type of *Xenia blumi*** SMF 44, Indonesia, Ternate island, 1894, coll. Kükenthal.

##### Description.

The holotype is 30 mm high and 10 mm wide at its base. The stalk splits into two branches. The first is 15 mm long, 6 mm wide at its base and 10 mm wide at its uppermost part; the second splits further into two branches, 15 and 7 mm long, each 5 mm wide at the base, and 10 and 7 mm wide at the upper part, respectively. Polyp’s body is up to 5 mm long and the tentacles up to 5 mm long. The pinnules are mostly arranged in two rows, with an occasional third row. There are 12–16 pinnules in the outermost row, 1 mm long and 0.14 mm wide, with a 0.2 mm space between adjacent pinnules. Sclerites are very scarce in all parts of the holotype; they are *Ovabunda*-type spheroids, measuring 0.012–0.028 × 0.018–0.036 mm in diameter (n = 35 sclerites). Occasionally, two sclerites are fused, reaching 0.050 mm in maximal diameter. SEM micrographs of sclerites were obtained only from the additional material due to low density of sclerites in the holotype. Reinicke (1997) recorded non-pulsating polyps for the species. The ethanol preserved holotype is light beige.

##### Remarks.

**Figure 10.**
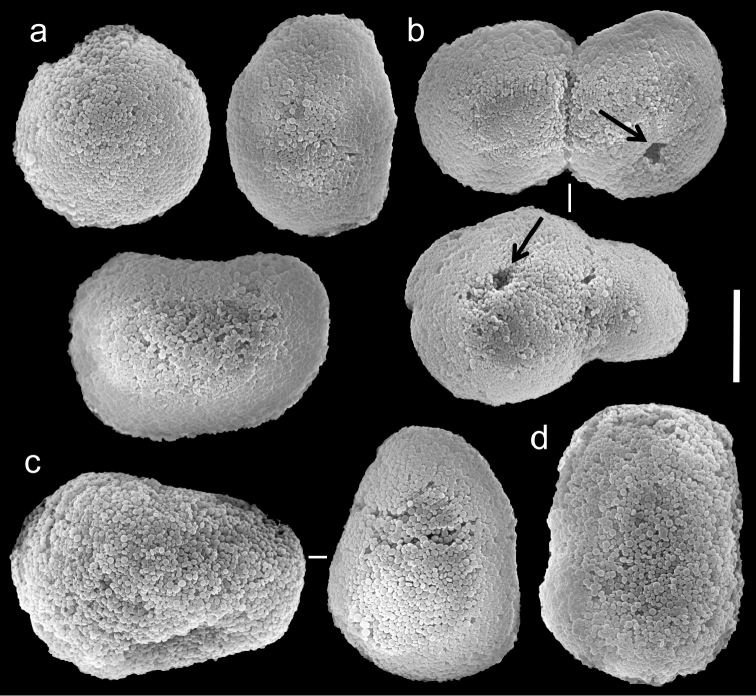
Scanning electron micrographs of polyp sclerites of *Ovabunda crenata* (Reinicke, 1997) (RMNH Coel. 23517). **a** Regular sclerites **b** Fused sclerites **c** Egg-shaped sclerites **d** Rectangular sclerite. Arrows indicate surface dents. Scale bar 10 µm.

RMNH Coel. 23517 is similar in size to the holotype. Polyp’s body is up to 2 mm long, with 2 mm long tentacles, mostly bearing two rows of 1 mm long and 0.1 mm wide slender pinnules on each of the tentacle sides, and, rarely, a third row. The outermost row features 12–15 pinnules, up to one pinnule-width apart. Sclerites are of the *Ovabunda*-type, varying in shape from regular (Fig. 10a), egg-shaped (Fig. 10c) and more rectangular (Fig. 10d), ranging 0.016–0.026 × 0.028–0.040 mm (n = 25 sclerites) in diameter. Occasionally, two sclerites are fused, reaching 0.047 mm in maximal diameter (Fig. 10b).

Reinicke (1997) noted under the description of *Xenia crenata* sp. n. (p. 41): “?*Xenia blumi* Schenk 1896; Gohar 1940: 98, Plate 5” and “*Xenia blumi* Schenk 1896 sensu Gohar – Reinicke 1995: 42, Fig. 31”. Reinicke (1997) also noted: “?*Xenia viridis* Schenk 1896; Benayahu 1990: 115 (listed)”. The description of *Xenia blumi* according to Gohar (1940) does not match *Ovabunda crenata* features. Examination of the types of *Xenia blumi* and *Xenia viridis* during the current study (see Table 1) revealed them to have platelet-shaped sclerites composed of dendritic rods, and to differ from *Ovabunda crenata*.

##### Conclusions.

The original description of the holotype indicated three rows of pinnules, whereas the present examination revealed mostly two rows, with an indication of a third one. There is agreement between our findings and the original description regarding the number of pinnules in the outermost row of the tentacles (12–16 *vs.* 12–15, respectively). Sclerites correspond to the original description in size but are *Ovabunda*-type spheroids; therefore, it is concluded that the species should be assigned to *Ovabunda*.

##### Similar species.

*Ovabunda crenata* is most similar to *Ovabunda biseriata*. Although they both have overlapping number of pinnules in the outermost row, 12–16 and 13–16, respectively, *Ovabunda biseriata* has two rows of pinnules and *Ovabunda crenata* occasionally presents a third row. They both have non-pulsating polyps in live colonies.

##### Distribution.

Red Sea: Gulf of Aqaba, Sudan.

#### Ovabunda faraunensis

(Verseveldt & Cohen, 1971)

http://species-id.net/wiki/Ovabunda_faraunensis

Fig. 11

Xenia faraunensis Verseveldt & Cohen, 1971: 62, table 1; Benayahu 1990 table 1 listed only; Reinicke 1997: 35, figs 3b, 9a-b, plates 2-4, 7, 22.
Ovabunda faraunensis ; Alderslade 2001: 51; Aharonovich and Benayahu 2011.

##### Material.

**Holotype:** HUJ I. Co. 140, northern Red Sea, Gulf of Aqaba, opposite Gezirat Fara’un (Coral Island) (29°27'46.95"N, 34°51'27.38"E), 18 m, 15 October 1969, coll. Y. Cohen.

##### Description.

**Figure 11.**
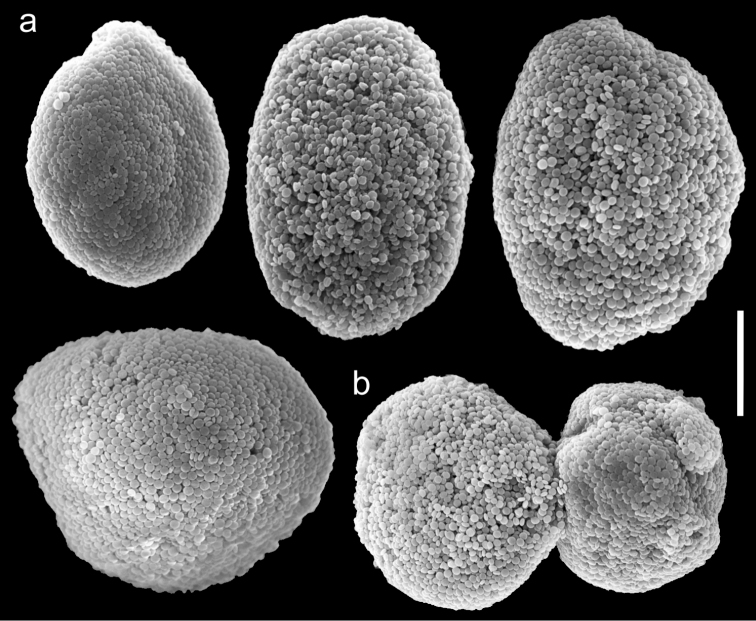
Scanning electron micrographs of polyp sclerites of *Ovabunda faraunensis* (Verseveldt & Cohen, 1971) holotype (HUJ I. Co. 140). **a** Regular sclerites **b** Fused sclerite. Scale bar 10 µm.

The holotype is 16 mm high; its stalk is 6-10 mm long, 4 mm wide at the base and 7 mm wide at its uppermost part. Polyp’s body is up to 2-4 mm long and the tentacles 3-5 mm long, featuring two rows of pinnules on each side with 17-24 pinnules in the outermost row. The sclerites are *Ovabunda*-type spheroids (Fig. 11a), measuring 0.013–0.028 × 0.019–0.033 mm in diameter (n = 46 sclerites). Occasionally, two sclerites are fused, reaching up to 0.044 mm in maximal diameter (Fig. 11b). Verseveldt and Cohen (1971) recorded non-pulsating polyps for this species. Colour of the ethanol-preserved holotype is beige.

##### Remarks.

At the time of examination the holotype was dry, and therefore precise dimensions of the pinnules could not be obtained. The original description (Verseveldt and Cohen 1971: 62) indicated that: “The colony is 25 mm high. The stem is 15 mm high and 5–6 mm wide at the base, then narrows to 3–4 mm and widens again to 7 mm or more at the beginning of the polyparium. The anthocodiae are up to 10 mm long... the tentacles are 5–6.5 mm long”. It is evident that the dimensions of the dried holotype are smaller than those of the original. The other features of the holotype recorded correspond to the original description, including two rows of pinnules, 17–24 pinnules in the outermost row, and sclerite diameter up to 0.044 mm (*vs.* 17–23 and 0.042 mm, in the original description). Reinicke (1997) presented a SEM micrograph of a single sclerite of *Ovabunda faraunensis* which later led Alderslade (2001) to assign it to the genus *Ovabunda*.

##### Conclusions.

The current examination of the holotype (Fig. 11) along with the examination of the holotype by Aharonovich and Benayahu (2011), further confirmed the previous assignment.

##### Similar species.

*Ovabunda faraunensis* is most similar to *Ovabunda arabica*. Although they both have two rows of pinnules and both have non-pulsating polyps in living colonies, the number of pinnules in the outermost row ranges from 17–24 in *Ovabunda faraunensis* compared to 24–29 in *Ovabunda arabica*.

##### Distribution.

Red Sea: Gulf of Aqaba, Sudan (see Reinicke 1997).

#### Ovabunda gohari

(Reinicke, 1997) comb. n.

http://species-id.net/wiki/Ovabunda_gohari

Fig. 12

Xenia gohari Reinicke, 1997: 30–31, plate 18.

##### Material.

**Holotype:** RMNH Coel. 23435 and a **paratype:** RMNH Coel. 23436 northern Red Sea, Gulf of Aqaba, 10 km south to Aqaba, Nature Reserve (Agaba) (29°26'29.03"N, 34°58'0.50"E), 10 m, November 1991 and 3 December 1991, respectively; coll. G. B. Reinicke.

##### Description.

**Figure 12.**
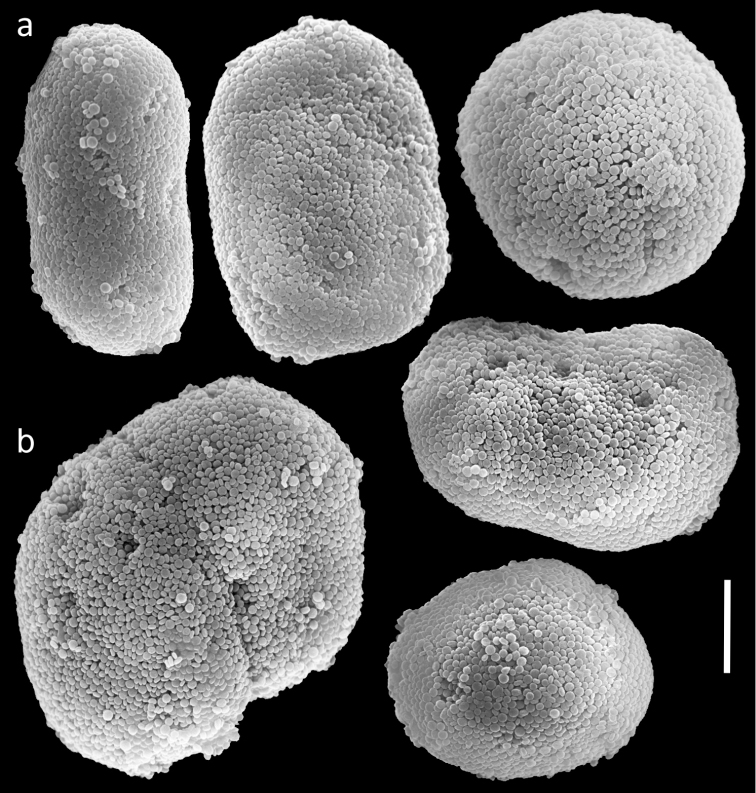
Scanning electron micrographs of polyp sclerites of *Ovabunda gohari* (Reinicke, 1997) paratype (RMNH Coel. 23436). **a** Regular sclerites **b** Fused sclerite. Scale bar 10 µm.

The holotype is 25 mm high; the stalk is 15 mm long, 10 mm wide at its base and 20 mm wide at its uppermost part and is divided into two. Polyp’s body reaches 5 mm and the 11 mm long tentacles mostly feature a single row bearing 19–22, 3 mm long and 0.2 mm wide pinnules. The gap between the pinnules is up to twice their width; occasionally a second row exists and then the outermost row bears 19–22 pinnules. The gap between adjacent pinnules in the outmost row is almost twice their width, as in the case of a single row. The sclerites are *Ovabunda*-type spheroids, measuring 0.028–0.045 mm in diameter (Fig. 12).

The paratype is 18 mm long. Its stalk is divided into two branches, 8 mm and 4 mm long; each branch is 3 mm wide at its base, and 5 and 4 mm wide at the uppermost part, respectively. Polyp’s body is up to 3 mm long, and tentacles 5–8 mm long, each have one row of 18–21 pinnules on each side; some small polyps have only 11–13 pinnules. The pinnules are up to 3 mm long and 0.24 mm wide at the base and the gap between the pinnules is up to twice their width. The sclerites (Fig. 12a) measure 0.015–0.035 × 0.028–0.055 mm in diameter (n = 30 sclerites). Occasionally, two sclerites are fused, measuring up to 0.060 mm in maximal diameter; the fusion of sclerites can be partial or almost complete (Fig. 12b). The sclerites are more abundant at the base of the pinnules along the tentacle midline compared to their distal part, and similarly at the aboral side of the tentacles compared to their oral side. The original description indicated non-pulsating polyps in live colonies. The ethanol-preserved colony is light yellowish, beige.

##### Remarks.

The current study confirmed the number of rows of pinnules and number of pinnules of the original description. Sclerite dimensions of the latter are larger than our findings, probably due to measurements that included fused sclerites.

##### Conclusions.

The sclerites are *Ovabunda*-type spheroids and justify assignment to that genus.

##### Similar species.

*Ovabunda gohari* is most similar to *Ovabunda faraunensis*. Although they both have overlapping number of pinnules in the outermost row, 18–22 and 17–24, respectively, *Ovabunda gohari* has mostly one row of pinnules and *Ovabunda faraunensis* presents two rows of pinnules. They both have non-pulsating polyps in live colonies.

##### Distribution.

Red Sea: Gulf of Aqaba, Sudan (see Reinicke 1997).

#### Ovabunda hamsina

(Reinicke, 1997)

http://species-id.net/wiki/Ovabunda_hamsina

Fig. 13

Xenia hamsina Reinicke, 1997: 49-50; figs 20a–b, plate 30.
Ovabunda hamsina ; Janes 2008: 610–611.

##### Material.

**Holotype:** RMNH Coel. 23904, Sudanese Red Sea, Sanganeb Atoll, off Port Sudan, reef flat (19°21'33.81"N, 37°19'37.66"E), 6 April 1991, coll. G. B. Reinicke; **paratypes:** RMNH Coel. 23902, same data as above, April 1991; RMNH Coel. 25906, same locality SW corner, 15 m; RMNH Coel. 23553, Sudanese Red Sea, Sanganeb Atoll, lagoon slope near TQ II, 12 m, October 1992, coll. G. B. Reinicke; **additional material:** RMNH Coel. 23552, same locality, W-slope, TQ IV, 12 m, April 1991, coll. G. B. Reinicke; RMNH Coel. 25903, same locality, SE corner, reef flat; RMNH Coel. 25905 SW corner, 15 m; RMNH Coel. 23907, near southern jetty, 10 m; all April 1991, all coll. G. B. Reinicke; RMNH Coel. 23908, Indian Ocean, Madagascar, 1960, coll. M. Cherbounier, MNHN Oct.A.1993.16; **holotype of *Xenia grasshoffi*** SMF 2616, northern Red Sea, Gulf of Aqaba, Elat, 1 January 1968, coll. Grasshoff M.

##### Description.

**Figure 13.**
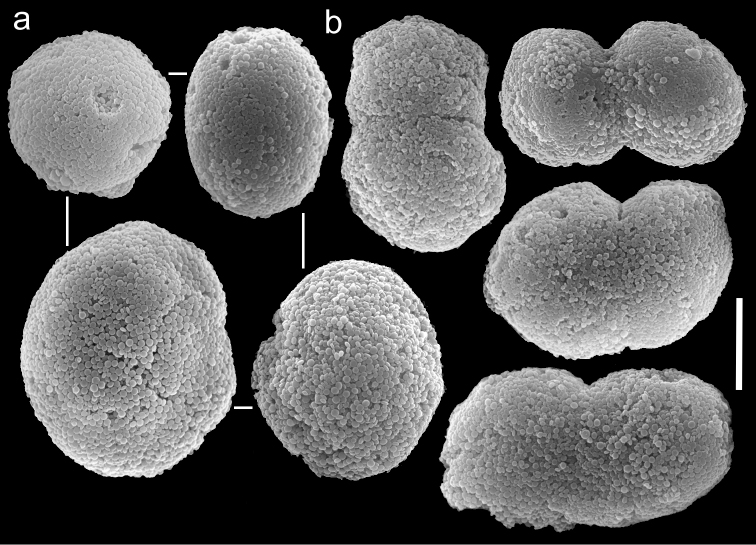
Scanning electron micrographs of polyp sclerites of *Ovabunda hamsina* (Reinicke, 1997) holotype (RMNH Coel. 23904). **a** Regular sclerites **b** Fused sclerites. Scale bar 10 µm.

The holotype is 25 mm high and its stalk is 18 mm long, 15 mm wide at its base and 12 mm wide at its uppermost part. Polyp’s body reaches up to 2 mm long; tentacles 5 mm long featuring three rows of 1 mm long and 0.16 mm wide pinnules, 19-21 in the outermost row. An incomplete fourth row is occasionally present. The pinnules are separated by a two-pinnule width space. The sclerites are *Ovabunda*-type (Fig. 13a), 0.015–0.025 × 0.016–0.035 mm in diameter (n = 25). Occasionally, two sclerites are partially fused, reaching 0.034 mm in maximal diameter (Fig. 13b). The tentacles of the paratypes (RMNH Coel. 25902, 23533, 25906) bear three rows of pinnules on each side, with 19–22, 19–21 and 17–22 pinnules in the outermost row, respectively, and all feature *Ovabunda*-type sclerites. The original description indicated non-pulsating polyps in live colonies. The ethanol-preserved colony is light beige.

##### Remarks.

The colonies RMNH Coel. 25905, 23907, 23908 feature three rows of pinnules with 17–22 pinnules in the outermost row. The tentacles of RMNH Coel. 23552 have four rows of pinnules and 18–21 pinnules in the outermost row; RMNH Coel. 25903 has three rows of pinnules with an indication of a fourth row, and 19–22 pinnules in the outermost row. All colonies feature *Ovabunda*-type sclerites.

Janes (2008) described *Ovabunda hamsina* from the Seychelles and the current findings confirm the assignment of *Ovabunda hamsina* to the genus *Ovabunda* based on sclerite microstructure.

Reinicke (1997) noted under the description of *Xenia hamsina* sp. n. (p. 49): “*Xenia* aff *grasshoffi* Reinicke, 1995: 59, Fig. 33”. The type of *Xenia grasshoffi* was examined by us (see Table 1), and found to have the type of sclerites found in *Xenia* and clearly different from *Ovabunda hamsina*.

**Similar species.** *Ovabunda hamsina* is most similar to *Ovabunda ainex*. Although they both have overlapping number of pinnules in the outermost row, 17–22 and 15–20, respectively, *Ovabunda ainex* has three rows of pinnules and *Ovabunda hamsina* presents three and sometimes four rows of pinnules. They both have non-pulsating polyps in live colonies.

**Distribution.** Sudanese Red Sea, Madagascar, Seychelles (see Janes 2008).

#### Ovabunda impulsatilla

(Verseveldt & Cohen, 1971)

http://species-id.net/wiki/Ovabunda_impulsatilla

Figs 14 ,15 ,16

Xenia impulsatilla Verseveldt & Cohen, 1971: 59–60, table 1; Verseveldt 1974: 2 listed only; Benayahu 1990, listed only; Reinicke 1997: 32, plate 19.
Xenia miniata Reinicke, 1997: 39–40 figs 17a-b, plate 24, **syn. n.**
Ovabunda aldersladei Janes, 2008: 613–614, figs 9–10, **syn. n.**
Ovabunda impulsatilla ; Janes 2008: 611–613, fig. 8; Aharonovich and Benayahu 2011.

##### Material.

**Holotype:** HUJ I Co. 84 northern Red Sea, Gulf of Aqaba, near Solar Pond (Sinai), (29°25'44.43"N, 34°49'50.31"E), 2 m, 15 August 1969, Coll. Y. Cohen. Eight colonies on a sponge, one of them is the holotype. **Additional material: the holotype of *Xenia miniata*:** RMNH Coel. 23514, Sudanese Red Sea, Sanganeb Atoll, 20 km off Port Sudan, W-slope, TQ IV, (19°21'33.81"N, 37°19'37.66"E), 12 m, March 1991; **paratypes of *Xenia miniata*:** RMNH Coel 25412, RMNH Coel 25413, details as above, RMNH Coel. 25411, same location, SW-corner slope, TQ I, 12 m, March 1991, coll. G. B. Reinicke; RMNH Coel. 6848, northern Red Sea, gulf of Suez, El Tur (28°14'10.99"N, 33°36'51.06"E), 6 July 1969, coll. L. Fishelson; RMNH Coel. 6847, same details; RMNH Coel. 8938, same details, Abu Durbah (28°28'27.56"N, 33°19'30.13"E); **the holotype of *Ovabunda aldersladei*** RMNH Coel. 38681, Indian Ocean, Seychelles, northern coast of Bird Island (03°42'S, 55°12'E), <30 m, 21 December 1992, Tyro expedition; type of ***Xenia ternatana*** SMF 43, Indonesia, Ternate island, 1894, coll. Kükenthal; **type of *Xenia garciae*** BML 1921.11.18.1, Indian Ocean, Chagos Archipelago, coll. Diego Garcia; **RMNH Coel. 8938** Red Sea, Gulf of Suez, Abu Zanima; **RMNH Coel. 6847**, Red Sea, Gulf of Suez, El Tur, 6 July 1969, coll. L. Fishelson; **RMNH Coel. 6848**, same location, misidentified as *Xenia miniata*

##### Description.

**Figure 14.**
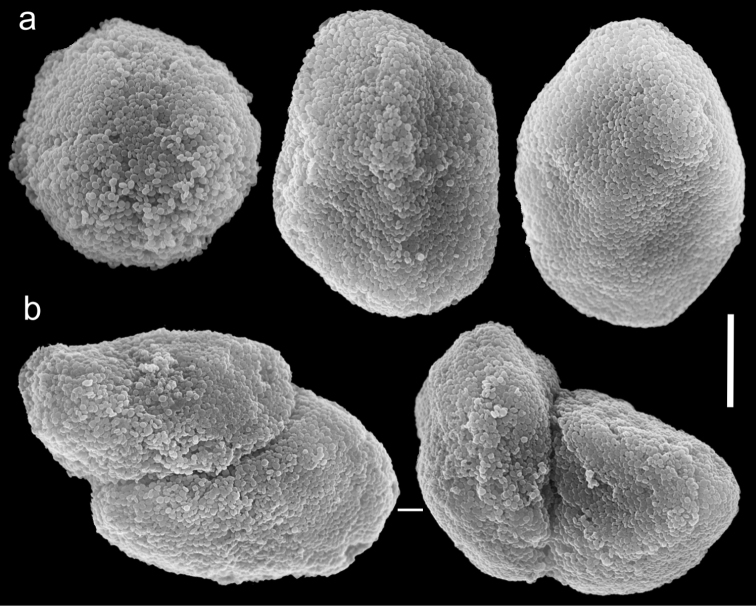
Scanning electron micrographs of polyp sclerites of *Ovabunda impulsatilla* (Verseveldt & Cohen, 1971) holotype (HUJ I Co. 84). **a** Regular sclerites **b** Fused sclerites. Scale bar 10 µm.

The now dry holotype is 18 mm high. The stalk is divided into two branches, 10 and 8 mm long, 6 and 4 mm wide at the base, and 8 and 4 mm wide at the uppermost part, respectively. Polyp’s body is up to 2–2.5 mm long, and tentacles up to 1.5–2 mm, featuring two rows of pinnules on each side, with 8–11 pinnules in the outermost row. The sclerites are*Ovabunda*-type (Fig. 14a) and are almost evenly distributed in all parts of the colony, measuring 0.013–0.028 × 0.019–0.039 mm in diameter (n = 36 sclerites). Occasionally, two sclerites are fused, measuring up to 0.045 mm in maximal diameter (Fig. 14b). The original description stated that polyp pulsation in this species occurs in live colonies. The ethanol-preserved colony is light brown.

##### Remarks.

At the time of examination the type was dry and, therefore, the dimensions of the pinnules are lacking and the current measurements do not reflect the original ones given by Verseveldt and Cohen (1971), who stated that “The colonies are 8–15 mm high and 10–15 mm wide. Three to four branches arise from a short stem 1–2 mm high. These branches, 2–3 mm high and 3–4 mm wide... The anthocodiae are 2 mm high and 1.2 mm wide, and tentacles are up to 2 mm long”. We encountered three colonies, one with a branched stalk and two with undivided stalks. It is possible that they originally belonged to a colony that disintegrated. The current findings in general agree with the original description, although the maximal diameter of sclerites noted in the original description is larger than our findings (0.030 *vs.* 0.044 mm, respectively). A colony from the Seychelles was also assigned to *Ovabunda impulsatilla* by Janes (2008). The SEM micrographs of the holotype sclerites given by Aharonovich and Benayahu (2011), along with the current ones (Fig. 14), confirm that the species should be assigned to the genus *Ovabunda*.

**Figure 15.**
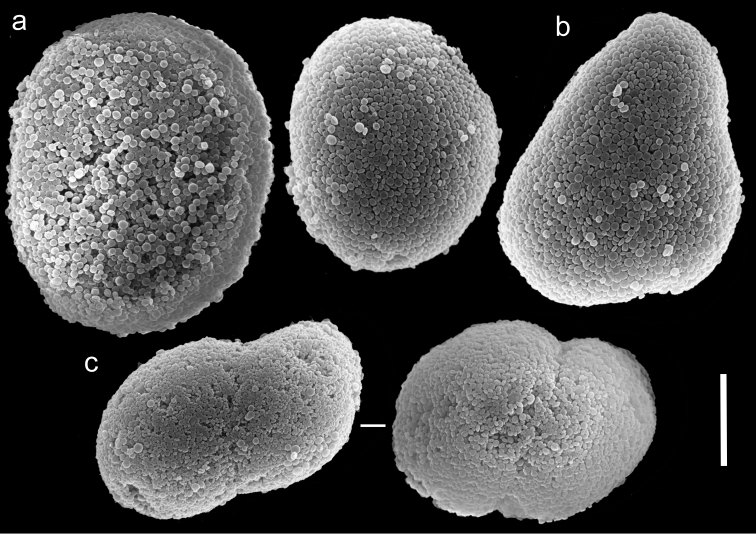
Scanning electron micrographs of polyp sclerites of *Xenia miniata* Reinicke, 1997 holotype (RMNH Coel. 23514). **a** Regular sclerites **b** Pear-shaped sclerite **c** Fused sclerites. Scale bar 10 µm.

The holotype of *Xenia miniata* (RMNH Coel. 23514) features two rows of pinnules on each side of the tentacles, with 8–13 pinnules in the outermost row. The sclerites are *Ovabunda*-type, some regular (Fig. 15a), some pear-shaped (Fig. 15b) measuring 0.017–0.034 × 0.022–0.053 mm in diameter (n = 30 sclerites). Occasionally, two sclerites are fused, reaching 0.059 mm in maximal diameter (Fig. 15c). The paratypes of that species (RMNH 25411, 25412, 25413) feature tentacles with two rows, 11–13 pinnules in the outermost row and *Ovabunda*-type of sclerites, measuring 0.018–0.030 × 0.024–0.034 mm in diameter (n = 24 sclerites). Although the original description of *Xenia miniata* indicated three rows of pinnules in the holotype and paratypes, we found only two rows. The original description of the species indicated non-pulsating polyps in live colonies. Colour of the ethanol preserved colony is beige. Consequently, we conclude that *Xenia miniata* should be synonymised with *Ovabunda impulsatilla*.

We also examined additional colonies that were identified by Reinicke (1997) as *Xenia miniata*. Specimen RMNH Coel. 6848 has two rows of pinnules, with 12–14 pinnules in the outermost row; its sclerites are *Ovabunda*-type, reaching up to 0.051 mm in maximal diameter. Based on sclerite size, number of pinnule rows and number of pinnules in the outermost row, this specimen should be reassigned to *Ovabunda biseriata*. Specimen RMNH Coel. 6847 has two rows of pinnules, with 10–11 pinnules in the outermost row; and its sclerites are also of the *Ovabunda*-type, reaching up to 0.045 mm in maximal diameter. RMNH Coel. 8938 has two rows of pinnules, but with only 8–9 in the outermost row; its sclerites are *Ovabunda*-type, reaching up to 0.047 mm in maximal diameter. Based on the number of rows of pinnules on the tentacles, the number of pinnules in the outermost row, and the size and microstructure of sclerites, the latter two colonies also belong to *Ovabunda impulsatilla*.

**Figure 16.**
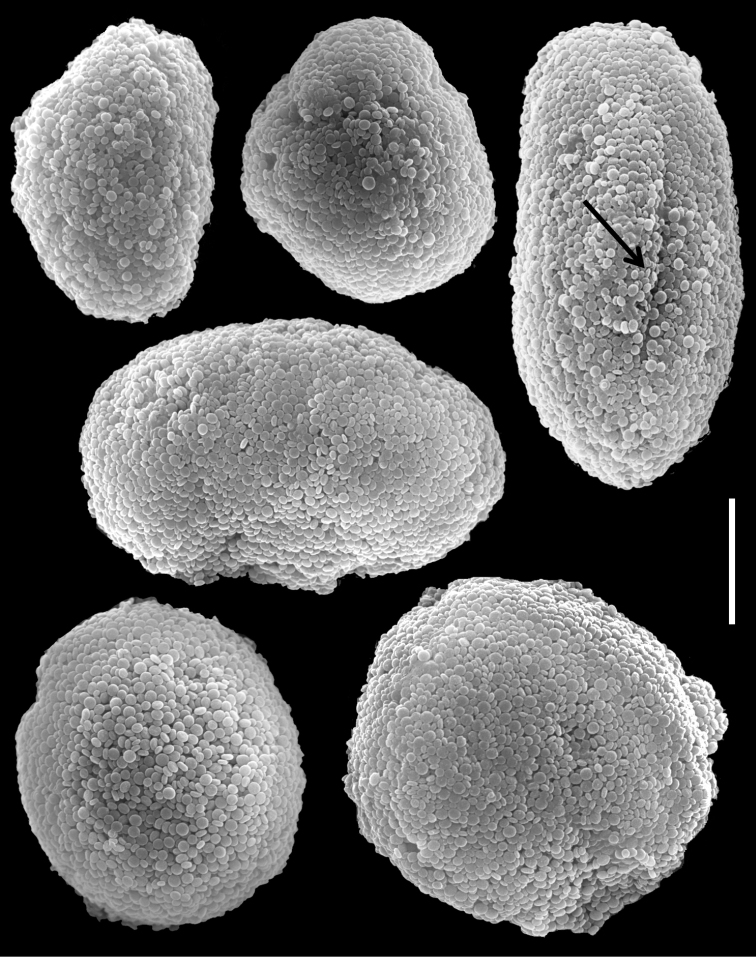
Scanning electron micrographs of polyp sclerites of *Ovabunda aldersladei* Janes, 2008 holotype (RMNH Coel. 38681). Arrow indicates surface crest. Scale bar 10 µm.

Our measurements of the dimensions of the holotype of *Ovabunda aldersladei* (RMNH Coel. 38681) agree with those of the original description. It features 3 mm long polyp’s body, 2 mm long tentacles, with two rows of pinnules on each side, and 8–12 pinnules in the outermost row. The densely set pinnules are up to 0.6 mm long and 0.2–0.3 mm wide and with almost no gap between adjacent pinnules. The sclerites are *Ovabunda*-type spheroids (Fig. 16), measuring 0.012–0.030 × 0.018–0.042 mm in diameter (n = 46 sclerites). Janes (2008) did not mention polyp pulsation for that species. The ethanol-preserved colony is light beige. The features of the holotype match the original description of *Ovabunda aldersladei*, except for sclerite size, which was found to be larger in our examination (up to 0.042 mm *vs.* up to 0.026 mm). The sclerites are indeed *Ovabunda*-type (Fig. 16), similar to those depicted by Janes (2008: fig. 10). Based on similarity between *Ovabunda aldersladei* and *Ovabunda impulsatilla* in number of rows (two in both), number of pinnules in the outermost row (8–12 and 8–11, respectively), and the size and microstructure of the sclerites it is concluded that the junior species *Ovabunda aldersladei* should be synonymized with *Ovabunda impulsatilla*, giving priority to the latter.

Reinicke (1997) noted under the description of *Xenia miniata* n.sp. (p. 39): “*Xenia ternatana* Schenk, 1896; Cohn 1908: 238 (ZMB 4991)” as well as: “nec *Xenia ternatana*; Kükenthal 1913: 8 (partim, NHMW C.16618)”. These two colonies were not examined in the current study. However, the type of *Xenia ternatana* was examined (see Table 1) and found to have *Xenia* type sclerites. Moreover, Reinicke 1997 noted: “*Xenia garciae*; Verseveldt 1970: 210 (RMNH Coel. 6846–6848); Verseveldt 1974: 2 (RMNH Coel. 8934, 8935, 8938); Reinicke 1995: 37, Fig. 30”. Several of the above mentioned colonies were found to be *Ovabunda impulsatilla* (see above), whereas the type of *Xenia garciae* presents *Xenia* type sclerites and thus is not a synonym of *Ovabunda impulsatilla*.

##### Similar species.

*Ovabunda impulsatilla* is most similar to *Ovabunda biseriata*. Although they both have two rows of pinnules and non-pulsating polyps in living colonies, the numbers of pinnules in the outermost row ranges from 8–11 in *Ovabunda impulsatilla* and 13–16 in *Ovabunda biseriata*.

**Distribution.** Red Sea: Egypt, Sudan; Seychelles.

#### Ovabunda macrospiculata

(Gohar, 1940)

http://species-id.net/wiki/Ovabunda_macrospiculata

Figs 17 ,18 ,19

Xenia macrospiculata Gohar, 1940: 96–98; Benayahu 1990 table 1 listed only; Reinicke 1997: 42, plates 1–3, 29.
Ovabunda macrospiculata ; Alderslade 2001: 51, figs 29–30.
Xenia macrospiculata Not ; Verseveldt 1971: 64–65, fig. 39.

##### Material.

**Neotype:** ZMTAU Co 25635, northern Red Sea, Gulf of Suez, Shag Rock (27°47'1.48"N, 33°59'23.17"E), <5m, 11 July 1986, coll. Y. Benayahu. **Paratypes: ZMTAU** Co 35789 (field number A69) northern Red Sea, Gulf of Aqaba, Underwater Restaurant (29°32'49.43N, 34°57'14.51"E), 5 m, 3 May 2010, coll. A. Halász; ZMTAU Co 35790 (field number A70) northern Red Sea, Gulf of Aqaba, The Interuniversity Institute of Marine Sciences in Eilat (IUI) (29°30'6.54"N, 34°55'4.44"E), 22 m, 4 May 2010, coll. A. Halász; ZMTAU Co 35791 (field number A72), collection details as above, 9 m.

##### Description.

**Figure 17.**
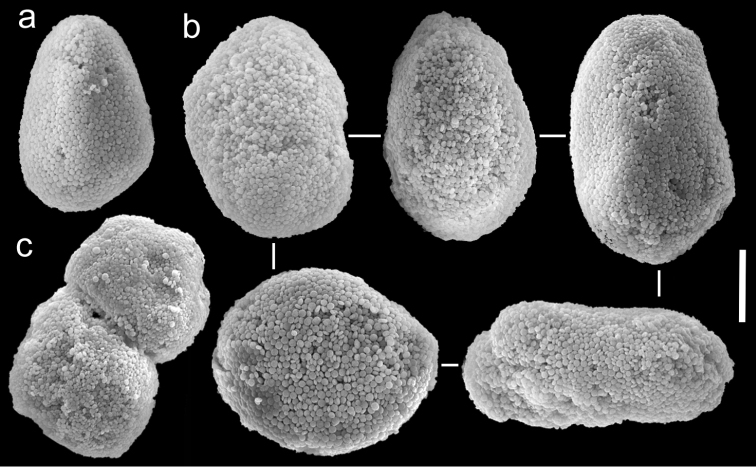
Scanning electron micrographs of polyp sclerites of *Ovabunda macrospiculata* (Gohar, 1940) neotype (ZMTAU Co 25635). **a** Pear-shape sclerite **b** Regular sclerites **c** Fused sclerite. Scale bar 10 µm.

The neotype is 20 mm high. Its stalk is 10 mm wide at its base, splitting into three branches, 7, 5 and 5 mm long; 5, 4 and 4 mm wide at the base and 10, 6 and 7 mm wide at the uppermost part, respectively. Polyp’s body reaches up to 2 mm and tentacles up to 5 mm, bearing three, occasionally two, rows of pinnules. The pinnules are 1 mm long and 0.2 mm wide, with less than a pinnule-width space between them, and there are 15–17 pinnules in the outermost row on each side of the tentacle. The sclerites are *Ovabunda*-type, both regular and pear-shaped, measuring 0.015–0.025 × 0.023–0.041 mm (n = 34, Fig. 17a, b). Occasionally two sclerites are fused, reaching 0.041 mm in maximal diameter (Fig. 17c).

**Figure 18.**
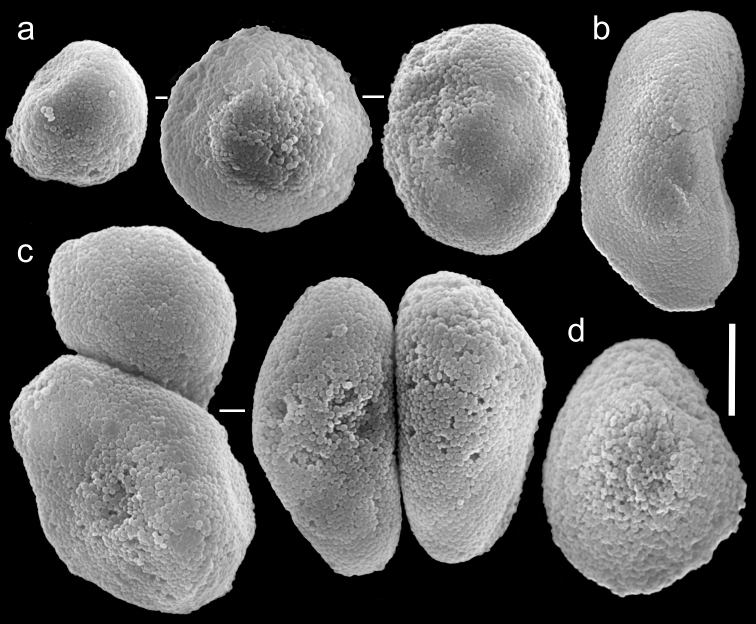
Scanning electron micrographs of polyp sclerites of *Ovabunda macrospiculata* (Gohar, 1940) paratype (ZMTAU Co 35790). **a** Regular sclerites **b** Irregular sclerite **c** Fused sclerites **d** Pear-shaped sclerite. Scale bar 10 µm.

**Figure 19.**
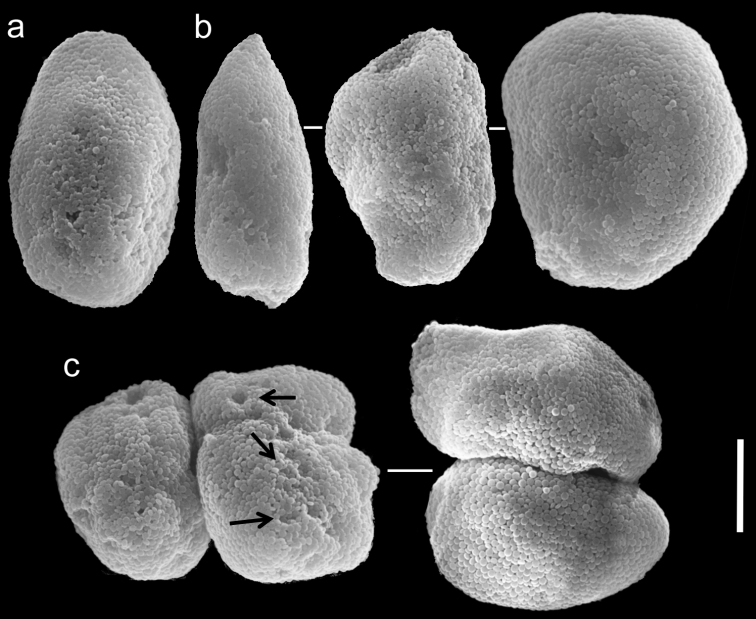
Scanning electron micrographs of polyp sclerites of *Ovabunda macrospiculata* (Gohar, 1940) paratype (ZMTAU Co 35791). **a** Regular sclerite **b** Irregular sclerites **c** Fused sclerites. Arrows indicate surface dents. Scale bar 10 µm.

The other specimens (ZMTAU Co 35789, 35790, 35791) are of similar size to the neotype; all with three rows of pinnules aligned on both sides of the tentacles, and 14–18, 14–17 and 14–17 pinnules in the outermost row, respectively. Their sclerites are of the *Ovabunda*-type and vary in shapes from regular to irregular, pear-shaped or fused. Their size ranges through 0.016–0.025 × 0.023–0.043 mm; 0.014–0.03 × 0.019–0.043 mm (Fig. 18) and 0.015–0.027 × 0.022–0.046 mm (Fig. 19) in diameter, respectively (n = 21 for each colony).

##### Remarks.

*Xenia macrospiculata* was originally described by Gohar (1940) from Ghardaqa, Egyptian Red Sea, as having pulsating tentacles, bearing three, occasionally two, rows of pinnules, with 12–16 pinnules in the outermost row (and 10–14 pinnules on the middle row, 0–10 on the oral one). That study did not indicate the museum in which the type was deposited. The last author of the current study searched in the museums listed in the Methods and found no trace of it; over time this type was probably lost. The designation of a neotype in this revision is thus necessary. The purpose of the designation is to clarify the species’ taxonomic status and its assignment to *Ovabunda*. Although the sclerites were described quite accurately in the original description (0.024–0.036 mm in diameter, and “spicules fused in pairs”), SEM micrographs of the sclerites are essential as in the other species of the revision.

The neotype was collected in proximity to the collection site of the original specimen collected by Gohar. The neotype is located at ZMTAU, and available upon request for future examination.

*Ovabunda macrospiculata* was also described by Verseveldt (1971: 64–65, fig. 39) from Nosy Be, Madagascar, referring to three and sometimes four rows of pinnules with 12–16 pinnules in the outermost row, and sclerites 0.020–0.042 mm in maximal diameter. Examination of RMNH Coel. 6702 from that study revealed that its tentacles have mostly four, rarely three rows of pinnules, and 14–16 pinnules in the outermost row (Reinicke 2013, pers. comm.). During our study the sclerites were examined and they are indeed *Ovabunda*-type, measuring up to 0.043 mm in maximum diameter, while fused sclerites are 0.055 mm in diameter. Based on the number of rows of pinnules and the number of pinnules at the outermost row, we conclude that it does not agree with *Ovabunda macrospiculata*.

##### Similar species.

*Ovabunda macrospiculata* is most similar to *Ovabunda ainex*. Although they both have three rows of pinnules, the numbers of pinnules in the outermost row ranges from 14–18 in *Ovabunda macrospiculata* compared to 15–20 in *Ovabunda ainex*. The major difference between them is that *Ovabunda macrospiculata* presents pulsating polyps in live colonies and *Ovabunda ainex* does not.

##### Distribution.

Red Sea: Gulf of Aqaba, southern tip of Sinai Peninsula.

#### Ovabunda verseveldti

(Benayahu, 1990)

http://species-id.net/wiki/Ovabunda_verseveldti

Fig. 20

Xenia verseveldti Benayahu, 1990: 115–116, fig. 2, table 1; Reinicke 1997: 29–30, plate 16.
Ovabunda verseveldti ; Alderslade 2001: 49.

##### Material.

Holotype: ZMTAU Co 26048 and four paratypes: ZMTAU Co 31625 northern Red Sea, Gulf of Aqaba, Dahab (28°30'34.21"N, 34°31'18.26"E), 1 m, 9 November 1979, coll. Y. Benayahu.

##### Description.

**Figure 20.**
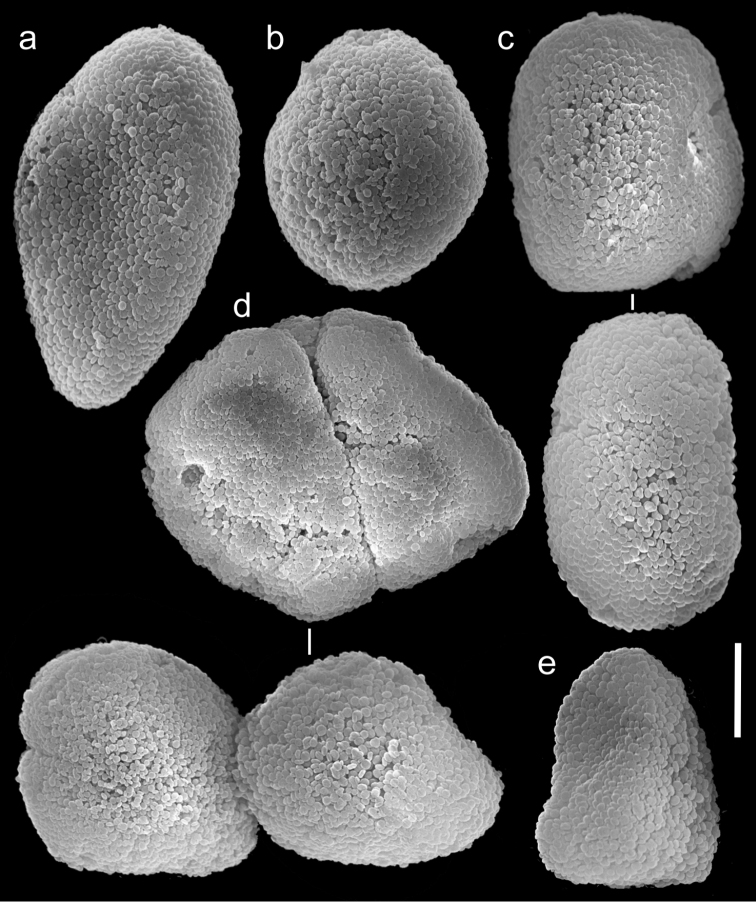
Scanning electron micrographs of polyp sclerites of *Ovabunda verseveldti* (Benayahu, 1990) holotype (ZMTAU Co 26048). **a** Egg-shaped sclerite **b** Regular sclerite **c** Rectangular sclerites **d** Fused sclerites **e** Irregular sclerite. Scale bar 10 µm.

The holotype is 18 mm high; its stalk is 9 mm long, 5 mm wide at its base and 13 mm wide at its uppermost part. Polyp’s body is 1–4 mm long, and the tentacles 6 mm long, bearing 1.8 mm long and 0.2 mm wide pinnules separated from each other by a small gap, less than one pinnule width. A single row of 14–18 pinnules is aligned on each side of the tentacles. There are numerous sclerites in all parts of the colony, densely packed in the pinnules and scarce in the mid-line of the tentacles’ oral side. The sclerites are *Ovabunda*-type, varying in shape from regular spheroids (Fig. 20b) to egg-shaped (Fig. 20a) and more rectangular forms (Fig. 20c), measuring 0.014–0.033 × 0.022–0.046 mm in diameter (n = 45). Rarely, two sclerites are fused, reaching a diameter of up to 0.051 mm (Fig. 20d).

The four paratypes (ZMTAU Co 31625) are smaller than the holotype, 12–17 mm high; the stalk is 10–15 mm long, 4–6 mm wide at the stalk base and 6–8 mm wide at the upper part. Polyp’s body is up to 5 mm long, and tentacles up to 3 mm long, featuring one row of 12–17 pinnules on each side. The pinnules are 1.2 mm long and 0.2 mm wide at their base, densely set in each row, almost touching each other. The sclerites are *Ovabunda*-type, measuring 0.018–0.030 × 0.022–0.049 mm in diameter (n = 38). Occasionally, two sclerites are fused, reaching a maximum size of 0.049 mm. The original description did not mention polyp pulsation. The ethanol-preserved colonies are light brown.

##### Conclusions.

The features of the holotype and paratypes agree with the original description of the species. The species was assigned by Alderslade (2001) to *Ovabunda* (see Introduction) and this is confirmed in the current study.

##### Similar species.

*Ovabunda verseveldti* is most similar to *Ovabunda benayahui*. Although they both have one row of pinnules the numbers of pinnules in the outermost row ranges from 12–18 in *Ovabunda verseveldti* compared to 6–7 in *Ovabunda benayahui*.

##### Distribution.

Red Sea: Gulf of Aqaba, southern tip of Sinai Peninsula.

## Discussion

The current study revises the genus *Ovabunda* Alderslade, 2001, following examination of relevant type material. Examination of the types has confirmed the previous assignment of the following four species to this genus: *Ovabunda benayahui*, *Ovabunda faraunensis*, *Ovabunda hamsina*, *Ovabunda verseveldti* (see Alderslade 2001; Janes 2008; Aharonovich and Benayahu 2011). It synonymizes another three species: *Xenia crista* with *Ovabunda arabica*; *Ovabunda obscuronata* with *Ovabunda biseriata*; and *Xenia miniata* and *Ovabunda aldersladei* with *Ovabunda impulsatilla*. In addition, three *Xenia* species are assigned for the first time to this genus: *Xenia ainex*, *Xenia crenata* and *Xenia gohari*, and a neotype is designated for the lost type of *Ovabunda macrospiculata*, thus bringing the total number of *Ovabunda* species to 11. Moreover, examination of an additional 22 *Xenia* types (see Table 1) out of the 58 species listed in WoRMS (http://www.marinespecies.org/aphia.php?p=taxlist), including SEM of their sclerites, has furnished the required data for revision of that genus (Halászet al. in prep.).

The first taxonomic revision of the family Xeniidae was that by Kükenthal in 1902, who presented 26 *Xenia* species and five *Cespitularia*. That revision used various morphological features for the species description, such as colony dimensions, pinnule form, number of rows of pinnules and number of pinnules in the outermost row, and also specified the geographic distribution of each species. No details were given for the dimensions of the sclerites or their shape but occasionally their density or absence was noted. The subsequent revision by Hickson (1931a) listed 13 valid *Xenia* species, five *Cespitularia*, one *Heteroxenia*, and included a discussion of *Sympodium*. In that revision the number of rows of pinnules and the number of pinnules in the outermost row were presented for each species. Hickson pointed out the difficulty in counting the somewhat irregular rows along each edge of the tentacles, and referred to it as a general problem of the Xeniidae. In the current study we also encountered this difficulty (see Results: e.g., *Ovabunda crenata* and *Ovabunda hamsina*). Since Hickson’s study (1931a), no revision has been published on any genus of the family Xeniidae. The above-mentioned two revisions failed to establish a standardized template for species descriptions within the family, which most probably hindered further attempts to carry out such a revision.

Here we discuss the reliability of each of the characteristics used to diagnose species, according to the order they appear in the species descriptions for each species. Colony dimensions as presented in the current study might in part be determined by age (Verseveldt 1960: 242) and environmental factors (Meestert et al. 2001) and also change according to collection and preservation conditions. Such dimensions might also exhibit a wide variation within a given species (Gohar 1940), therefore we doubt their value in species-specific taxonomic descriptions. However, pinnule length and width and the gap between adjacent pinnules are suggested here as a diagnostic trait: e.g. in *Ovabunda arabica* and its synonym *Xenia crista*, which had 1.8–2.2 mm long, 0.2 mm wide pinnules, with one pinnule-wide gap between adjacent ones. Similar to other studies (e.g., Gohar 1940, Verseveldt and Cohen 1971, Benayahu 1990, and Reinicke 1997), the number of pinnule rows and the number of pinnules in the outermost row are suggested to be of major taxonomic importance for species distinction, and the key (see above) is mainly based on these features.

The application of SEM for octocoral taxonomic studies, and Xeniidae in particular, has significantly increased the resolution of sclerite imaging and led to the establishment of new taxa based on their microstructural features (e.g. *Ovabunda* Alderslade, 2001; *Fasciclia* Janes, 2008; *Yamazatum* Benayahu, 2010). In the current study, SEM revealed for *Ovabunda* species the full shape and size range of the spheroidal sclerites including fused ones. The latter type of sclerite is found in all species, although sometimes rare (as in the case of *Ovabunda verseveldti*). For each species the dimensions of the individual sclerites and the fused ones are presented, which together are necessary for future species description. The range of the smallest diameter of the single *Ovabunda* spheroids was found to be similar in all the types, ranging 0.026–0.035 mm (e.g., *Ovabunda ainex*: Fig. 3, *Ovabunda biseriata*: Fig. 8). Their maximal diameter is mostly 0.035–0.040 mm (e.g., *Ovabunda benayahui*: Figs 6–7; *Ovabunda macrospiculata*: Figs 17–19; *Ovabunda gohari* Fig. 12); and in some species, such as *Ovabunda gohari* (Fig. 12) and *Ovabunda verseveldti* (Fig. 20), they occasionally reach a larger size, up to 0.046–0.055 mm. It is important to emphasize that these larger sclerites are rare in the above-mentioned types, which mainly have sclerites within a range of 0.035–0.040 mm. In order to present the actual range of sclerite sizes we measured at least 20 sclerites from each colony, a standard that we recommend for future studies. The lack of such a detailed account in past studies has led to taxonomic errors, as in the establishment of *Ovabunda aldersladei* (Janes 2008: sclerite maximal diameter range 0.018–0.026 mm), which is in fact a synonym of *Ovabunda impulsatilla* (sclerite maximal diameter range 0.018–0.042, Fig. 16). It should also be noted that only high-quality and sharp SEM images of *Ovabunda* sclerites reveal the morphological features of the sclerites, composed of corpuscular microscleres that are diagnostic for that genus. The current findings reveal that *Ovabunda* species feature spheroids of various sizes (e.g., *Ovabunda benayahui*: Fig. 6, *Ovabunda gohari*: Fig. 12), and shapes, some of which are regular, spherical (e.g. *Ovabunda ainex*: Fig. 3a, *Ovabunda faraunensis*: Fig. 11a) and others less so (e.g., *Ovabunda macrospiculata*: Fig. 18b; *Ovabunda verseveldti*: Fig. 20e; *Ovabunda ainex*: Fig. 3c). The sclerites in the latter species can be pear-shaped, or egg-shaped, or more rectangular (e.g. *Xenia miniata*: Fig. 15b, *Ovabunda faraunensis*: Fig. 20a; *Ovabunda benayahui*: Fig. 7d, respectively). Occasionally the sclerites feature surface dents or crests (e.g., *Ovabunda crenata*: Fig. 10; *Ovabunda macrospiculata*: Fig. 19c; *Ovabunda ainex*: Fig. 3; *Ovabunda benayahui*: Fig. 7). There is no apparent correlation between these different shapes and the different species and therefore sclerite shape has not been included in the key (see above). Prior to the current study, SEM micrographs and detailed sizes of fused sclerites were never recorded in *Ovabunda* species, and were recognized by light microscopy only in the original descriptions of *Ovabunda macrospiculata* (Gohar 1940) and *Ovabunda obscuronata* (Verseveldt and Cohen 1971). In most studies the fused sclerites were erroneously considered to be large individual sclerites, giving them a size of up to 0.060 mm in maximal diameter (e.g. *Ovabunda benayahui*, *Ovabunda crenata*: Reinicke, 1997). In the case of the fused sclerites, the use of SEM has enabled the full range of shapes to be captured, including some that were almost fully fused and could not be detected using light microscopy (e.g., *Ovabunda benayahui*: Figs 6b and 7b; *Ovabunda gohari*: Fig. 12b), partially fused figure-eight shapes (*Xenia crista*: Fig. 5b; *Ovabunda impulsatilla*: Fig. 14b), or spheroids with only a medial narrowing (*Ovabunda hamsina*: Fig. 13b). Undoubtedly, when measuring sclerites under a light microscope, the existence of both individual spheroids and fused ones should be taken into account. The occurrence of fused sclerites and their significance to the taxonomy of the genus and other xeniid genera should be further examined.

Polyp pulsation of living xeniid colonies was first noted by Lamarck (1816) (in: Kremien et al. 2013) and later by Hickson (1931a: 154) and Gohar (1940: 82–83). This feature was considered indicative for species identification. Recently, Kremien et al. (2013) found that such pulsation increases photosynthesis, which in absolute energy gain greatly surpasses the added metabolic cost. Since 1940 it has been noted in most descriptions of *Xenia* and *Heteroxenia* species (e.g. Gohar 1940, Verseveldt and Cohen 1971; Reinicke 1995, 1997). Among *Ovabunda* species the state of pulsation (absence/presence) in living colonies has been noted for all species (Gohar 1940, Verseveldt and Cohen 1971; Reinicke 1995, 1997), except *Ovabunda verseveldti*. As in previous studies, we consider the pulsation state of living colonies an important characteristic and we recommend recording it when collecting colonies. Hickson’s revision (1931a) and several later publications (e.g., Benayahu 1990; Reinicke 1997; Janes 2008) noted the color of the colonies. We doubt color is of taxonomic value, however, since it can change with preservation, and also depends on the type and density of the symbiotic zooxanthellae (Berner et al. 1987, Siebeck et al. 2006). We have also noted that sclerite density and distribution affect tissue coloration (Reinicke 1997: 18).

**Figure 21.**
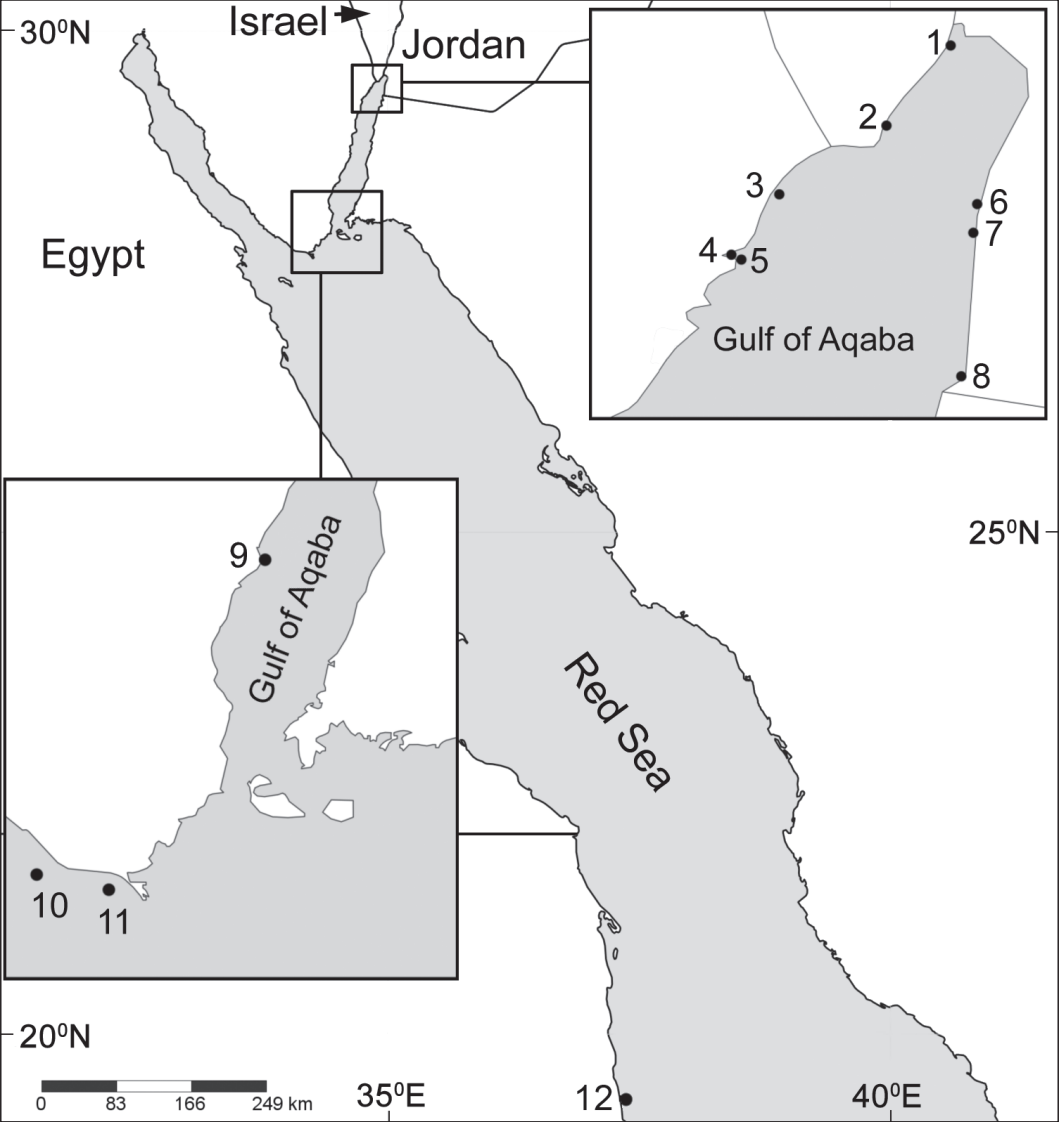
Distribution map of type localities of *Ovabunda* species. Areas of small rectangles are represented in respective large ones: **1** Underwater Restaurant, Eilat **2** The Interuniversity Institute of Marine Sciences (IUI), Eilat **3** opposite Gezirat Fara’un **4** Solar pond **5** Marsa Murach **6** Marine Science Station, Jordan **7** Nature reserve, Jordan **8** Saudi Arabia border bay **9** Dahab **10** Shag Rock **11** Shaab el Utaf **12** Sanganeb Atoll.

The current study indicates that the Red Sea is the type locality of most *Ovabunda* species (Fig. 21). Some species, such as *Ovabunda hamsina*, *Ovabunda impulsatilla*, and *Ovabunda macrospiculata*, were also recorded in the West Indian Ocean (e.g., Madagascar and the Seychelles). The possibility that the genus has a wider distributional range is not excluded, and remains to be confirmed by re-examination of already collected material deposited in various collections, or of freshly collected material from throughout the Indo-Pacific basin.

Re-examination and appropriate re-descriptions of octocoral type material, as conducted in the current study, is highly important in an era of molecular phylogeny and increasing phylogeographic studies, despite the difficulty or inability to extract DNA from the types themselves. This kind of comprehensive study based mainly on type material is a critical first step in the process of understanding phylogenetic relationships among species and genera, and their ecology. Due to similar morphologies in the case of *Xenia* and *Ovabunda*, further analysis is needed in order to reveal their radiation, especially in regions where they have a sympatric distribution. There is also a need to validate the current *Ovabunda* species, through an integrated taxonomic effort, combining molecular genetic evidence of species boundaries, ecological, and reproductive differences.

## Supplementary Material

XML Treatment for Xeniidae

XML Treatment for Ovabunda ainex

XML Treatment for Ovabunda arabica

XML Treatment for Ovabunda benayahui

XML Treatment for Ovabunda biseriata

XML Treatment for Ovabunda crenata

XML Treatment for Ovabunda faraunensis

XML Treatment for Ovabunda gohari

XML Treatment for Ovabunda hamsina

XML Treatment for Ovabunda impulsatilla

XML Treatment for Ovabunda macrospiculata

XML Treatment for Ovabunda verseveldti
